# Key Stratification of Microbiota Taxa and Metabolites in the Host Metabolic Health–Disease Balance

**DOI:** 10.3390/ijms24054519

**Published:** 2023-02-24

**Authors:** Alfonso Torres-Sánchez, Alicia Ruiz-Rodríguez, Pilar Ortiz, Margarita Aguilera

**Affiliations:** 1 Department of Microbiology, Faculty of Pharmacy, Campus of Cartuja, University of Granada, 18071 Granada, Spain; 2 Institute of Nutrition and Food Technology “José Mataix” (INYTA), Centre of Biomedical Research, University of Granada, 18016 Granada, Spain; 3 Department of Biochemistry and Molecular Biology II, Faculty of Pharmacy, Campus of Cartuja, University of Granada, 18071 Granada, Spain; 4 Instituto de Investigación Biosanitaria (IBS), 18012 Granada, Spain

**Keywords:** microbiota, taxa, metabolites, detoxification, pathways

## Abstract

Human gut microbiota seems to drive the interaction with host metabolism through microbial metabolites, enzymes, and bioactive compounds. These components determine the host health–disease balance. Recent metabolomics and combined metabolome–microbiome studies have helped to elucidate how these substances could differentially affect the individual host pathophysiology according to several factors and cumulative exposures, such as obesogenic xenobiotics. The present work aims to investigate and interpret newly compiled data from metabolomics and microbiota composition studies, comparing controls with patients suffering from metabolic-related diseases (diabetes, obesity, metabolic syndrome, liver and cardiovascular diseases, etc.). The results showed, first, a differential composition of the most represented genera in healthy individuals compared to patients with metabolic diseases. Second, the analysis of the metabolite counts exhibited a differential composition of bacterial genera in disease compared to health status. Third, qualitative metabolite analysis revealed relevant information about the chemical nature of metabolites related to disease and/or health status. Key microbial genera were commonly considered overrepresented in healthy individuals together with specific metabolites, e.g., *Faecalibacterium* and phosphatidylethanolamine; and the opposite, *Escherichia* and Phosphatidic Acid, which is converted into the intermediate Cytidine Diphosphate Diacylglycerol-diacylglycerol (CDP-DAG), were overrepresented in metabolic-related disease patients. However, it was not possible to associate most specific microbiota taxa and metabolites according to their increased and decreased profiles analyzed with health or disease. Interestingly, positive association of essential amino acids with the genera *Bacteroides* were observed in a cluster related to health, and conversely, benzene derivatives and lipidic metabolites were related to the genera *Clostridium*, *Roseburia*, *Blautia*, and *Oscillibacter* in a disease cluster. More studies are needed to elucidate the microbiota species and their corresponding metabolites that are key in promoting health or disease status. Moreover, we propose that greater attention should be paid to biliary acids and to microbiota–liver cometabolites and its detoxification enzymes and pathways.

## 1. Introduction

Gut microbiota is considered a complex ecosystem with a wide array of microorganisms linked to host health. Multiple studies suggested that the structure and composition of the gut microbiota in metabolic-related diseases, such as atherosclerosis, colitis, diabetes, hyperlipidemia, hypertension, metabolic syndrome, non-alcoholic fatty liver disease (NAFLD), non-alcoholic steatohepatitis (NASH), obesity, and steatosis, exhibit significant changes compared to healthy individuals and that those changes are related to host physiopathology. In this context, the analysis and description of trends in microbial populations associated with disease and health status become a key issue to elucidate possible signatures of metabolic-related diseases.

The gut microbiota of patients with metabolic-related diseases shows differences at different taxonomic levels. Many studies showed that *Parabacteroides, Bifidobacterium, Oscillospira,* and *Bacteroides* were decreased in patients with obesity [1,2,3,4,5,6,7,8,9,10,11,12,13]. Moreover, *Faecalibacterium* and *Bifidobacterium* were decreased [14,15,16,17,18,19,20,21] and species from Lactobacillaceae family [22] and *Blautia* were increased [7,13,19,20,21,22,23,24,25,26,27] in diabetic patients. Other metabolic diseases related to intestinal diseases seem to be related to increased *Escherichia* and decreased *Faecalibacterium* [28,29,30,31,32,33,34,35,36,37].

Recently, the combination of metagenomics and metabolomics has received extensive attention due to the growing number of studies that establish positive and negative correlations between gut microbiota taxa, metabolites, and health status. Therefore, future studies will contribute to elucidate the essential role of gut microbiota in metabolite synthesis, metabolite modifications, and metabolic pathway regulations.

In this sense, metabolites such as short-chain fatty acids (SCFA), amino acids (AA), or bile acids (BA) can play a crucial role in maintaining metabolic functions or, on the contrary, they might be involved in disease development, such as choline derivatives in the case of cardiovascular diseases [38,39,40,41]. Metabolite influences are not restricted to the intestine and distribution to other physiological locations has been described through different axes, such as the gut–liver axis, in which the gut microbiota is related to liver diseases, including NAFLD, NASH, fibrosis, or liver cancer [42]. Gut microbiota partially impacts the host BA profile as it is involved in primary bile acid transformation into secondary free bile acids, such as deoxycholic acid, lithocholic acid, and ursodeoxycholic acid, contributing to the modulation of host total bile acid production [43].

The chemical structure of many endogenous compounds, including gut microbiota metabolites, can be modified, resulting in changes in their bioactivity and half-life [44]. This kind of modifications are related to the development of complex metabolic networks between host and gut microbiota, where final substances could be potentially more toxic than the original ones [45].

Traditional probiotics, mainly consisting of species from Lactobacillaceae and Bifidobacteria and a few from other genera, have been largely applied as a useful strategy in the context of clinical intervention in metabolic-related diseases [46,47]. However, the development of new procedures using Next Generation Probiotics (NGP) opens a new world of possibilities due to the beneficial effects that have already been described in murine models and, to a lesser extent, in humans. In this context, murine models show *Akkermansia muciniphila, Faecalibacterium prausnitzii, Bacteroides uniformis, Bacteroides acidifaciens, Clostridium butyricum,* and *Prevotella copri* as interesting microorganisms with potential applications in obesity [48,49,50,51,52,53], liver diseases [52,54,55,56,57,58,59], diabetes [48,49,50,51,52,53,58,60,61], colitis [62], and hyperlipidemia [53,58].

This work will contribute to finding out microbial and metabolite patterns and their correlation with diseases that have been studied independently or not yet extensively studied. Therefore, the principal aim of this work is to identify and describe the association between human gut microbiota taxa changes in metabolic-related diseases, incorporating the correlations with metabolites, and how they can modulate host health.

## 2. Results

### 2.1. Differential Microbiota Taxa Composition and Stratification According to Their Representation in Metabolic Diseases

#### 2.1.1. PRISMA Analysis

**Figure 1:**
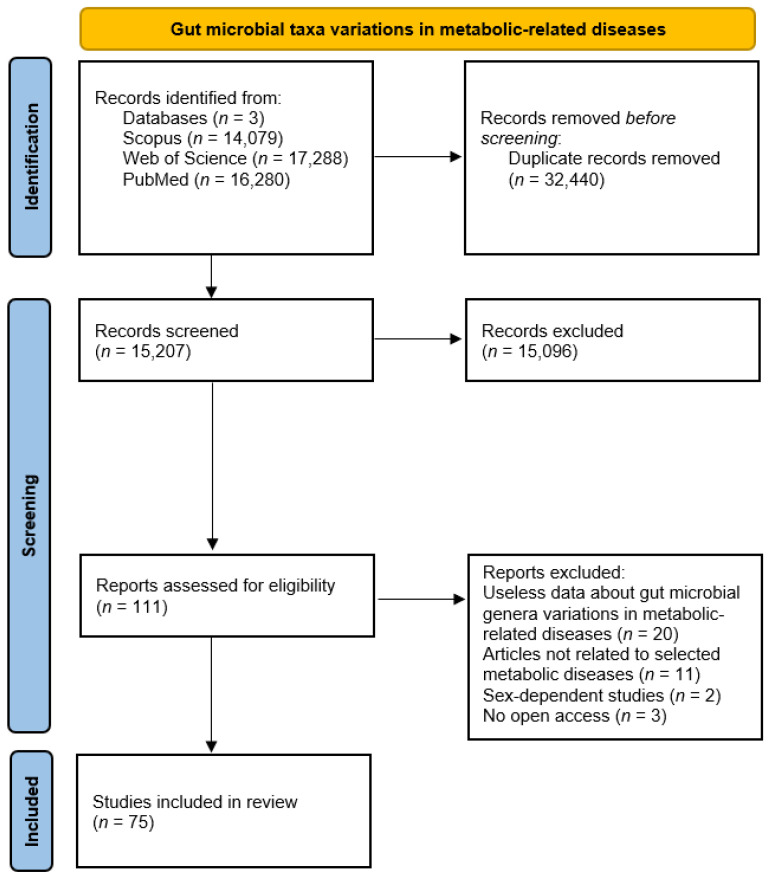
PRISMA diagram for gut microbial taxa changes in metabolic diseases.

Gut microbial taxa differences in diabetes, obesity, metabolic syndrome, and liver and cardiovascular diseases, highlight links between gut microbiota and host health status. In this context, Figure 1 summarizes updated and available information about gut microbial taxa changes in these metabolic-related diseases.

#### 2.1.2. Microbial Taxa Decreased in Patients Suffering from Metabolic-Related Diseases

Increased and decreased trends in gut microbiota taxa were assessed through an extensive literature search including information about metabolic diseases investigated by different authors. In this context, the approach we followed offered some drivers of specific changes in gut microbiota composition that could be related to host health.

**Figure 2:**
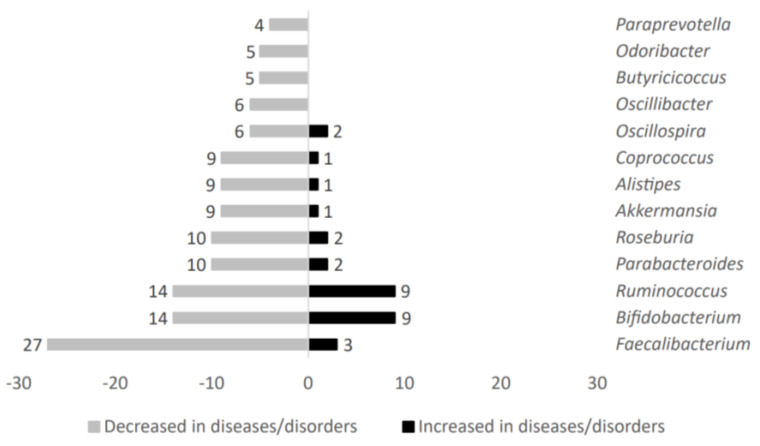
Analysis of main taxa stratified according to high representativeness in patients without metabolic-related diseases.

The analysis of 75 studies involving changes of the main taxa altered in patients suffering metabolic-related diseases disclosed 121 differentially abundant microbial genera (complete data are available in Supplementary Material S1). Figure 2 shows representative genera count value comparison obtained in metabolic diseases after microbial taxa variation analysis.

Gut microbiota genera such as *Oscillibacter*, *Butyricicoccus*, *Odoribacter*, and *Paraprevotella* were exclusively decreased in individuals affected by metabolic diseases. On the other hand, *Faecalibacterium*, *Bifidobacterium*, *Ruminococcus*, *Parabacteroides*, *Roseburia*, *Akkermansia*, *Alistipes*, *Coprococcus*, and *Oscillospira* were both decreased and increased in metabolic-related diseases. However, overall, these microbial genera showed a negative association with the metabolic diseases studied here.

#### 2.1.3. Microbial Taxa Increased in Patients Suffering Metabolic-Related Diseases

**Figure 3:**
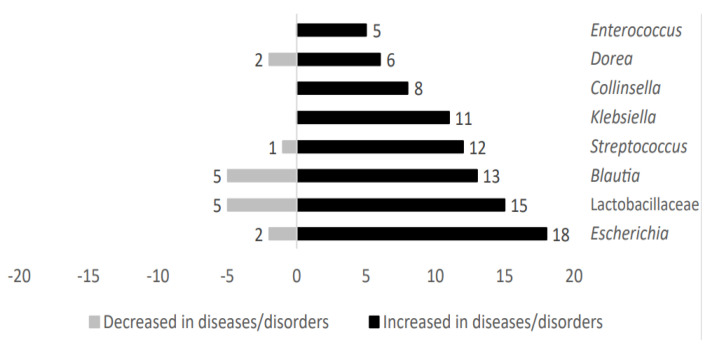
Analysis of main taxa stratified according to high representativeness in metabolic−related diseases patients.

Microbial genera such as *Klebsiella*, *Collinsella*, and *Enterococcus* were exclusively present in those cases in which individuals were affected by metabolic diseases. However, taxa belonging to *Escherichia*, Lactobacillaceae, *Blautia*, *Streptococcus*, and *Dorea* were also identified in patients without metabolic-related diseases. These microbial genera showed an upward trend in metabolic-related diseases studied here. Figure 3 shows the distribution of representative microbial taxa linked to metabolic-related diseases.

In a previous study exploring next generation probiotics for metabolic and microbiota dysbiosis linked to xenobiotic exposure [63], we tried the first approach to describe changes in gut microbial taxa associated to metabolic-related disease. As a result, potential associations between bacterial genera and metabolic diseases were described despite the lesser number of analyzed studies. In this case, Table 1 shows an expansion of the current knowledge available in this field, including the relevant information identified in the previous study.

### 2.2. Differential Microbial Metabolites and Stratification According to Their Representation in Metabolic Diseases

**Figure 4:**
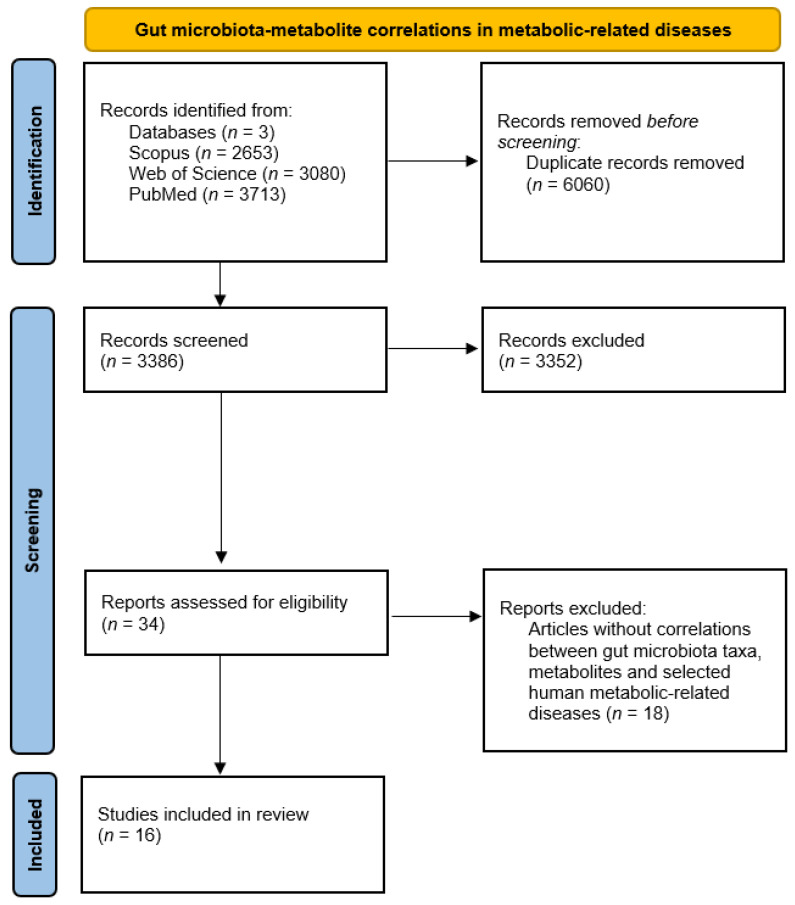
PRISMA diagram for gut microbiota–metabolite correlations and host status.

The analysis of the 16 selected studies involving correlations between gut microbiota taxa altered in patients suffering from metabolic diseases, metabolites, and host health status allowed us to shed light on potential critical pathways to modulate homeostatic processes (complete data are available in Supplementary Material S2 [103,104,105,106,107,108,109,110,111,112,113,114,115,116,117,118] Figure 4 summarizes available information about gut microbiota–metabolite correlations and host health status.

**Figure 5:**
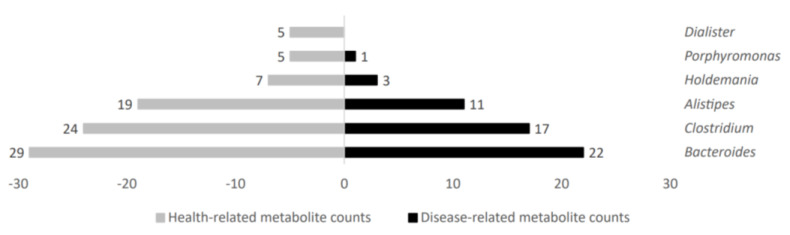
Health−related metabolite counts stratified according to gut microbiota taxa producers.

Several gut microbiota taxa showed a high metabolite count linked to disease or health status. In that regard, increased microbial metabolite counts in health status were obtained in gut microbiota genera such as *Holdemania, Porphyromonas,* and *Dialister*; further, they were also higher for *Bacteroides, Clostridium,* and *Alistipes*, but with more similar counts in both groups. Figure 5 shows representative genera differential values associated to health-related metabolite count analysis.

**Figure 6:**
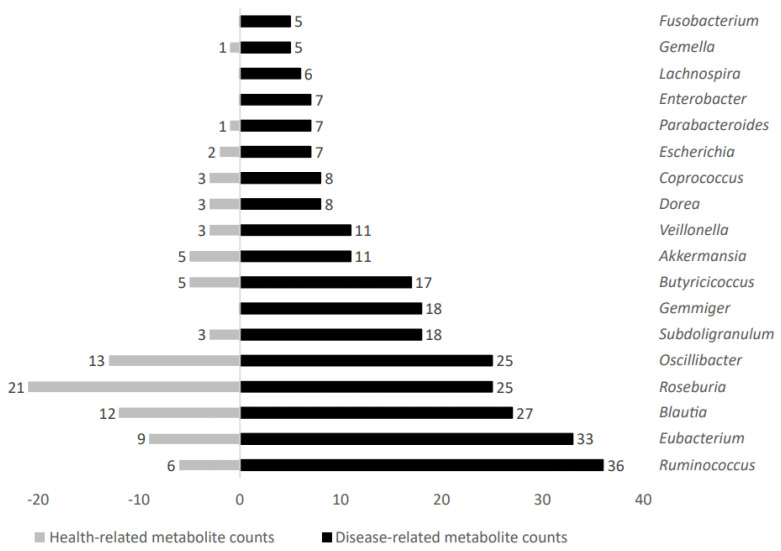
Disease−related metabolite counts stratified according to gut microbiota taxa producers.

Increased metabolite counts related to disease status were linked to gut microbiota taxa such as *Ruminococcus*, *Eubacterium*, *Blautia*, *Roseburia*, *Oscillibacter*, *Subdoligranulum*, *Gemmiger*, *Butyricicoccus*, *Akkermansia*, *Veillonella*, *Dorea*, *Coprococcus*, *Escherichia*, *Parabacteroides*, *Enterobacter*, *Lachnospira*, *Gemella*, and *Fusobacterium*. Figure 6 shows representative genera differential values associated to disease-related metabolite count analysis.

According to the total metabolites linked to disease and health status, 171 metabolites were associated with metabolic-related diseases; among these, 143 were exclusively associated with this group and 28 were shared with health status. Moreover, 63 metabolites were related to health status, and 35 were exclusively associated with this group. A qualitative metabolite analysis was performed considering total disease/health-related metabolites. Table 2 shows disease/health-related metabolites classified according to three main chemical groups: fatty acids and conjugates, amino acids and derivatives, and bile acids and derivatives.

**Figure 7:**
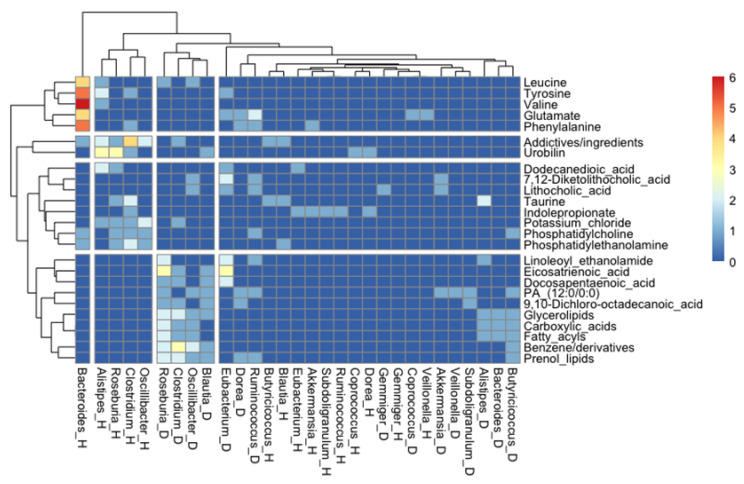
Heatmap showing the analysis where specific associations between a metabolite and a bacterial genus was found in a health and/or a disease stage (as indicated by “_H” or “_D”, respectively). For simplicity, only the representative genera and the most found metabolites were included.

A further association analysis of the number of studies where a specific association between a metabolite and a bacterial genus was found showed very interesting clustering patterns. For instance, butyrate-producer genera when present in a healthy status associated with bile acid metabolites and, to a lesser extent, with essential amino acids; however, when they are overrepresented in metabolic diseases, they are associated with lipid metabolism, clustering in two distinct groups. We also observed that essential amino acids clustered together, and they might have an important role for the metabolism of *Bacteroides* in health status, according to Figure 7.

## 3. Materials and Methods

We performed a comprehensive literature search covering the period from 1995 to November 2022 using Scopus, Web of Science, and PubMed databases, using the search strategies showed in systematic review and dividing this review into two main study issues: gut microbial taxa variations in metabolic-related diseases and gut microbiota–metabolite correlations in metabolic-related diseases.

Studies involving changes in gut microbial taxa in atherosclerosis, colitis, diabetes, hyperlipidemia, hypertension, metabolic syndrome, NAFLD, NASH, obesity, and steatosis and studies involving microbiota–metabolite correlations in metabolic-related diseases were assessed, screened, and selected according to PRISMA 2020 flow diagrams (Figure 1 and Figure 4) [111].

In the microbial taxa variation analysis, gut microbial taxa identified in selected studies were divided into two groups: decreased in metabolic-related diseases and increased in metabolic-related diseases, based on research findings. Metabolite counts were calculated for each microbial genus. To determine representative gut microbiota taxa, an arbitrary criterion was applied. Microbial genera were considered representative if the absolute frequency difference between decreased–increased counts was greater than three.

In the gut microbiota–metabolite correlation analysis, gut microbiota, microbial metabolites, and host status correlations were assessed. First, gut microbial genera were classified into increased in health status or increased in diseases, according to metabolite absolute frequencies displayed for each genus. Second, considering metabolites related to representative genera in health or disease status, a qualitative metabolite analysis was performed. Metabolites correlated with health or disease status were classified into three main groups: fatty acids and conjugates (FA), amino acids and derivatives (AA), and bile acids and derivatives (BA), according to PubChem and related chemical database classification. Furthermore, a bioinformatics analysis was performed to establish potential biomarkers, which revealed the association between specific disease/health balances. Heatmap shows the analysis where a specific association between a metabolite and bacterial genera was found in a health and/or a disease stage (as indicated by “_H” or “_D”, respectively). For simplicity, only the representative genera and the most found metabolites (metabolites that appeared least five times either associated with health or disease in the studies analyzed here) were included. First, we selected only the genera with more than 10 metabolites associated and then we kept only the metabolites that appeared at least five times, either associated with health or disease, in the studies analyzed here. Figure 7 shows the performance of R (version 4.1.1.) using the package “pheatmap” [112].

## 4. Discussion

There is a growing interest in the analysis of the gut microbiome and its metabolome [113,114]. However, integrating data from both fields to understand how gut microbiota, microbial metabolites, and host status are correlated not always provide concise information. Thus, it can hinder researchers in establishing clear links between the presence of a particular gut bacterial taxa and/or metabolites and disease or health status. This task is especially challenging in the context of searching gut microbial biomarkers that allow predicting future phenotypes or classifying individuals into disease and non-disease status. This is mainly due to the fact that contradictory results about microbial taxa abundance and metabolites related to disease or non-disease status can be found in the literature. In this case, this approach showed that *Faecalibacterium, Bifidobacterium, Ruminococcus, Parabacteroides, Roseburia, Akkermansia, Alistipes, Coprococcus, Oscillospira, Oscillibacter, Butyricicoccus, Odoribacter,* and *Paraprevotella* could represent a downregulated microbial cluster in metabolic-related disease patients and, on the contrary, *Escherichia,* species from Lactobacillaceae family, *Blautia*, *Streptococcus*, *Klebsiella*, *Collinsella*, *Dorea*, and *Enterococcus* cluster upregulation could be involved in metabolic-related disease status. Due to relevant information underlined by many authors and results obtained in this review, *Ruminococcus* and *Bifidobacterium*, as well as taxa belonging to Lactobacillaceae family, *Blautia*, and *Dorea* should be identified at the species level to establish similarities with the results already available in the microbiological databases.

According to metabolite absolute frequencies in disease and health status and representative gut microbiota taxa, we tried to search for possible trends between those elements and host physiopathology. When we compared representative metabolites and microbial taxa results, only *Alistipes*, from the down-regulated proposed cluster, showed high counts in both gut microbial taxa variation analysis and metabolite count analysis related to health. In the same way, *Escherichia, Blautia, Streptococcus, Collinsella, Dorea*, and *Enterococcus,* from the proposed upregulated cluster, showed high counts in both gut microbial taxa analysis and metabolite count analysis in disease/disorder group.

Following this approach, *Faecalibacterium* and *Akkermansia* genera [115,116], frequently described as key microorganisms related to health status, were decreased in metabolic-related diseases, indicating a possible relationship with health status. However, a link with disease status could be identified according to metabolite absolute frequencies described for both genera *Faecalibacterium* and *Akkermansia*. A similar result can be observed in other microorganisms frequently associated with metabolic diseases [117], where microbial taxa analysis showed links with obesity-related diseases. However, metabolite absolute counts showed links with health status.

Interestingly, preliminary data results derived from the biomarker search have demonstrated the positive association of essential amino acids with health in the genera *Bacteroides,* and conversely, benzene derivatives have been related to disease and the genera *Clostridium*. We also observed that lipid metabolites grouped several taxa overrepresented in diseases, but it will be necessary to determine the results to the species level.

These results showed which bacterial taxa of the gut microbiota and their derived metabolites could be related to host status manifestations. However, study limitations and lack of available data in some fields make it impossible to establish final and solid conclusions in this way.

Human health is not only affected by gut microbiota composition and its derived metabolites but also many exogenous and endogenous factors, which can also impact in genotypic and phenotypic manifestations. Recently, the holistic concept of the One Health approach and the exposome include multidisciplinary analysis of a complex reality that affect different but linked items [118]. Nowadays, solid evidence about specific microbial and metabolite signatures in cases of metabolic-related disease is still limited and more concrete information on the correlations between gut microbiota, gut metabolites, and host health status is needed. This synergic approach will lead to a better management of well-known microbiota–metabolic related diseases.

To increase the availability of scientific data on the interaction between gut microbiota taxa in different health contexts, metabolite synthesis, and metabolite modification and impact on the host health, integrated metagenome and metabolome analysis should be continually reviewed, since it seems to be a possible cornerstone involved in the determination of potential microbial and metabolite signatures related to physiological alterations.

## 5. Conclusions

Despite the existence of microbial taxa–metabolite-health correlations, there is no evidence of a clear gut microbiota and derived metabolite patterns into healthy or metabolic-related disease status that is able to predict or classify patients into one or the other.

Most of the taxa and metabolites did not show representative oscillations between disease and health groups, so bacterial genera with potential interest should continue to be monitored as new information on their abundance in metabolic-related disease appearance.

Implementation of the One Health holistic approach combined with exposome principles can provide new perspectives and evidence about how endogenous and exogenous substances interact with gut microbiota and microbial-derived substances and how the pull of interactions finally affects human homeostasis.

## Figures and Tables

**Table 1:** Changes in the main microbiota taxa found in patients suffering metabolic-related diseases.

Ref.	Sample Size and Clinical Traits	Gut Microbiota Taxa Modification
[1]	*n* = 42; HC *n* = 21; OB *n* = 21	↑ *Prevotella, Megamonas, Blautia,* and *Fusobacterium,* ↓ *Alistipes, Faecalibacterium, Oscillibacter, Clostridium IV, XIVa, Barnesiella, Gemmiger, Parabacteroides, Coprococcus, Ruminococcus,* and *Bifidobacterium* in OB
[2]	*n* = 51; HC *n* = 30; OB/OW *n* = 21	↑ Lactobacillus *, ↓ *Bifidobacterium* in OB/OW
[3]	*n* = 51; HC *n* = 23; OB/OW *n* = 28	↑ *Faecalibacterium, Phascolarctobacterium, Lachnospira, Megamonas,* and *Haemophilus,* ↓ *Oscillospira,* and *Dialister* in OB
[4]	*n* = 192; HC *n* = 25; OW *n* = 22; OB *n* = 145	↑ *Escherichia coli, Pseudomonas, Fusobacterium,* ↓ *Bifidobacterium* in OW/OB
[5]	*n* = 143; HC *n* = 56; OB *n* = 87	↑ *Enterococcus, Blautia, Sutterella, Klebsiella,* and *Collinsella,* ↓ *Bacteroides, Parabacteroides, Anaerotruncus,* and *Coprobacillus* in OB
[6]	*n* = 78; HC *n* = 36; OB *n* = 42	↓ *Bacteroides* in OB
[23]	*n* = 66; HC *n* = 27; OB *n* = 17; OBT2D *n* = 22	↑ *Staphylococcus* in OB; ↑ Lactobacillus * and *Escherichia* in T2D
[7]	OW *n* = 34; OB *n* = 23; AbOB *n* = 53; Dys *n* = 78; IFG *n* = 21; IGT *n* = 3; T2D *n* = 21; HT *n* = 34	↑ *Serratia* and *Prevotella,* ↓ *Oscillospira* in OW, OB, AbOB group; ↑ *Blautia* in T2D; ↑ *Prevotella* in HT
[8]	*n* = 58; HC *n* = 15; OB *n* = 18; OB NAFLD *n* = 25	↑ *Phascolarctobacterium, Phascolarctobacterium succinatutens, Klebsiella, Klebsiella pneumoniae, Kluyvera,* and *Kluyvera ascorbata,* ↓ Lactobacillus *, Oscillibacter, Ruminiclostridium, and *Parabacteroides johnsonii* in OB NAFLD; ↓ *Alistipes, Paraprevotella, Bacteroides clarus*, and *Odoribacter splanchnicus* in OB and OB NAFLD; ↓ *Helicobacter, Helicobacter pylori* in OB
[9]	*n* = 73; HC *n* = 20; OB NAFLD *n* = 36; OB Non-NAFLD *n* = 17	↑ Megasphaera, Lactobacillus *, and *Acidaminococcus,* ↓ *Oscillospira, Eubacterium,* and *Akkermansia* in OB NAFLD and OB Non-NAFLD; ↑ *Streptococcus,* ↓ *Blautia, Alkaliphilus,* and *Flavobacterium* in OB NAFLD
[10]	*n* = 115; HC *n* = 54; OB *n* = 8; NAFLD *n* = 27; NASH *n* = 26	↑ *Bradyrhizobium, Anaerococcus, Peptoniphilus, Propionibacterium acnes, Dorea,* and *Ruminococcus,* ↓ *Oscillospira* in NAFLD, NASH and OB vs. HC
[11]	*n* = 23; HC *n* = 10; NASH *n* = 13	↑ Lactobacillus * in (OB-NASH vs. LN-HC), (OB-NASH vs. OB-HC) and (OB-NASH vs. OW-NASH); ↑ *Lachnospira* in (OB-NASH vs. OB-HC); ↓ *Roseburia* in (OB-NASH vs. LN-HC) and (OB-NASH vs. OB-HC); ↓ *Bifidobacterium* in (OW-NASH vs. LN-HC); ↓ *Faecalibacterium* and *Ruminococcus* in (LN-NASH vs. LN-HC) and (LN-NASH vs. OB-HC); ↓ *Ruminococcus* in (LN-NASH vs. OB-NASH) and (LN-NASH vs. OW-NASH)
[64]	*n* = 106; HC *n* = 38; OB *n* = 68	↑ *Clostridium* in HT; ↑ *Bacteroides* in IGT
[12]	*n* = 119; OB *n* = 69; Mets *n* = 50	*↑ Intestinibacter, Saccharibacteria genera incertae sedis, Clostridium sensu stricto, Romboutsia, Terrisporobacter,* and Eggerthia, ↓ Rothia, Adlercreutzia, Parabacteroides, Paraprevotella, Alistipes, Bacteroides, Bilophila, Escherichia-Shigella, Lactobacillus *, Clostridium XIVa, Clostridium XIVb, Anaerotruncus, and *Phascolarctobacterium* in OB vs. Mets
[65]	*n* = 60; HC *n* = 20; OB T2D *n* = 40	↑ *Eubacterium coprostanoligenes* group, *Dialister*, and *Allisonella*, ↓ *Ruminococcus 2, Prevotella 9,* and *Escherichia-Shigella 9* in OB T2D
[14]	*n* = 1280; LN-NonT2D *n* = 633; OB-NonT2D *n* = 494; OBT2D *n* = 153	↓ *Akkermansia, Faecalibacterium, Oscillibacter,* and *Alistipes* in OB- NonT2D and OBT2D
[15]	*n* = 50; HC *n* = 15; T2D *n* = 14; DR *n =* 21	↑ *Klebsiella* and *Enterococcus,* ↓ *Faecalibacterium* and *Lachnospira* in T2D
[16]	*n* = 154; CN *n* = 73; T2DCI *n* = 81	↑ *Peptococcus,* ↓ *Bifidobacterium, Veillonella,* and *Pediococcus* in T2DCI
[66]	*n* = 291; HC *n* = 193; T2D *n* = 98	↑ *Peptostreptococcus, Eubacterium,* and *Prevotella,* ↓ *Anaerostipes, Ruminococcus, Clostridium, Epulopiscium, Cellulosilyticum ruminicola, Clostridium paraputrificum,* and *Clostridium butyricum* in T2D
[17]	*n* = 60; HC *n* = 40; T2D *n* = 20	↑ *Streptococcus, Fusobacterium,* and *Dorea,* ↓ *Parabacteroides, Bifidobacterium, Faecalibacterium,* and *Akkermansia* in T2D
[24]	*n* = 102; HC *n* = 35; pT2D *n* = 17; NewT2D *n* = 11; KnownT2D *n* = 39	↑ *Escherichia* and *Acidaminococcus,* ↓ *Sutterella* in KnownT2D; ↑ Megasphaera and Lactobacillus *, ↓ *Akkermansia*, *Blautia*, and *Ruminococcus* in NewT2D
[67]	*n* = 118; HC *n* = 59; T2D *n* = 59	↑ *Bifidobacterium* spp., ↓ *Bacteroides* spp. in T2D
[25]	*n* = 100; HC *n* = 35; T2D+ *n* = 49; T2D− *n* = 16	↑ *Coprococcus 1,* ↓ *Bacteroides* and *Prevotella* in T2D+ and T2D- vs. HC; ↑ *Parasutterella* in T2D+ vs. HC; ↑ *Blautia* and *Eubacterium hallii* group in T2D−vs. HC
[26]	*n* = 100; HC *n* = 50; T2D *n* = 50	↑ Lactobacillus *, ↓ C*lostridium leptum* and *Clostridium coccoides* in T2D
[18]	*n* = 36; HC *n* = 18; T2D *n* = 18	↓ *Faecalibacterium prausnitzii* in T2D
[19]	*n* = 36; HC *n* = 18; T2D *n* = 18	↑ Lactobacillus *, ↓ *Bifidobacterium* in T2D
[68]	*n* = 239; HC *n* = 54; HT *n* = 97; HL *n* = 96; T2D *n* = 162	↑ *Bifidobacterium* in HL, T2D, RISK1, and RISK2; ↑ *Collinsella* in HT, HL, T2D, RISK2, and RISK3; ↑ *Escherichia* in RISK3; ↓ *Alistipes* in HL
[27]	*n* = 98; HC *n* = 47; T1D *n* = 51	↑ *Blautia, Anaerostipes, Eubacterium hallii* group, *Dorea, Collinsella*, and *Klebsiella,* ↓ *Parabacteroides* and *Flavonifractor* in T1D
[69]	*n* = 29; HC *n* = 8; T1D at onset *n* = 8; T1D two years treatment *n* = 13	↑ *Bacteroides,* ↓ *Prevotella, Megamonas,* and *Acidaminococcus* in T1D at onset
[70]	*n* = 47; HC *n* = 7; T1D *n* = 22; T2D *n* = 18	↑ *Pseudomonas* and *Prevotella* in T1D and T2D vs. HC
[20]	*n* = 110; HC *n* = 40; T1D *n* = 21; T2D *n* = 49	↑ *Escherichia, Prevotella,* and Lactobacillus *, ↓ *Bacteroides, Roseburia,* and *Bifidobacterium* in T1D and T2D; ↓ *Faecalibacterium* in T1D vs. T2D and HC
[21]	*n* = 43; HC *n* = 13; T1D *n* = 15; MODY2 *n* = 15	↑ B*acteroides, Ruminococcus, Blautia, Veillonella, Streptococcus, Sutterella,* and *Enterobacter,* ↓ *Bifidobacterium* in T1D; ↑ *Prevotella* ↓ *Lachnospira, Roseburia, Anaerostipes,* and *Faecalibacterium* in T1D and MODY2
[71]	*n* = 60; HC *n* = 30; Metsyn patients *n* = 30	↑ *Clostridium leptum, Clostridium coccoides* group, and *Turicibacter* sp., ↓ *Butyricicoccus* sp., *Faecalibacterium prausnitzii*, and *Akkermansia muciniphila* in Mets
[72]	*n* = 655; MZ *n* = 306; DZ *n* = 74, Nontwin *n* = 275	↑ Lactobacillus *, Sutterella, Dorea, and *Methanobrevibacter,* ↓ P*arabacteroides, Bifidobacterium, Odoribacter, Akkermansia*, and *Paraprevotella* in Mets
[13]	*n* = 20; No Mets + NGT *n* = 4; No Mets + IFG *n* = 3; No Mets + IFG + IGT *n* = 1; Mets + IFG *n* = 4; Mets + IFG + IGT *n* = 4; Mets + T2D *n* = 4	↑ *Ruminococcus, Dorea, Blautia,* and *Oscillospira* in OB, Mets, IFG, IFG + IGT, and T2D
[28]	*n* = 41; HC *n* = 20; UC *n* = 21	↓ *Ruminococcus* and *Faecalibacterium prausnitzii* in UC
[29]	*n* = 20; HC *n* = 10; UC *n* = 10	↑ *Escherichia-Shigella, Peptostreptococcus, Bacillus*, and *Veillonella,* ↓ *Akkermansia, Faecalibacterium,* and *Bifdobacterium* in UC
[30]	*n* = 42; HC *n* = 14; UC *n* = 28	↑ *Streptococcus, Escherichia-Shigella, Romboutsia, Clostridium sensu stricto, Enterococcus,* and *Citrobacter,* ↓ *Faecalibacterium, Agathobacter, Dorea, Ruminococcus*, *Prevotella, Alistipes, Parabacteroides,* and *Butyricicoccus* in UC
[73]	*n* = 53; HC *n* = 23; UC *n* = 12; PSC *n =* 11; PSC + UC *n =* 7	↑ *Bifidobacterium* in UC
[31]	*n* = 24; HC *n* = 12; CD *n* = 6; UC *n* = 6	↑ *Clostridium ramosum, Escherichia coli, Fusobacterium nucleatum,* and *Ruminococcus gnavus,* ↓ *Eubacterium rectale,* and *Faecalibacterium prausnitzii* in UC
[32]	*n* = 58; HC *n* = 29; UC *n* = 22; CD *n* = 7	↓ *Bacteroides, Faecalibacterium prausnitzii, Prevotella* spp., and *Methanobrevibacterium* spp. in IBD
[33]	*n* = 42; HC *n* = 13; CD *n* = 15; UC *n* = 14	↑ *Abiotrophia*, *Pseudoramibacter, Eubacterium,* and *Escherichia,* ↓ *Butyricicoccus, Mitsuokella, Haemophilus,* and *Victivallis* in CD; ↑ *Granulicatella, Peptostreptococcus, Schwartzia, Capnocytophaga, Escherichia, Janthinobacterium, Campylobacter, Actinomyces, Eggerthella,* and *Corynebacterium,* ↓ *Holdemania, Lachnobacterium, Megamonas, Mitsuokella, Alistipes, Butyricimonas, Prevotella, Desulfovibrio, Oxalobacter, Pyramidobacter,* and *Victivallis* in UC; ↑ *Pseudoramibacter Eubacterium, Desulfovibrio,* and *Slackia,* ↓ *Butyricicoccus, Moryella, Staphylococcus, Capnocytophaga, Haemophilus, Janthinobacterium, Cardiobacterium, Lautropia, Lupinus, Shewanella,* and *Corynebacterium* in CD/UC
[34]	*n* = 155; Non-IBD *n* = 34; CD *n* = 68; UC *n* = 53	↑ Unclassified *Roseburia* species in CD and UC; ↑ *Bifidobacterium breve* and *Clostridium symbiosum* in UC; ↑ *Blautia producta, Lactobacillus gasseri, Enterococcus faecium, Clostridium clostridioforme, Ruminococcus gnavus,* and *Escherichia coli* in CD
[74]	*n* = 1087; HC *n* = 290; IBD *n* = 512; CRC *n =* 285	↑ *Bacteroides* in IBD
[35]	*n* = 68; HC *n* = 48; IBD *n* = 20	↑*Bifidobacterium, Ruminococcus gnavus* group, *Streptococcus*, and *Blautia,* ↓ *Faecalibacterium, Subdoligranulum, Parabacteroides*, and *Paraprevotella* in IBD
[36]	*n* = 30; HC *n* = 8; DD *n* = 4; IBS *n* = 3; UC *n* = 5; CD *n* = 10	↑ *Dialister* spp. And *Faecalibacterium prausnitzii* in IBS; ↑ *Bacteroides fragilis, Dialister spp.,* and *Roseburia* spp. ↓ *Clostridium difficile* in UC vs. HC; ↑ *Parabacteroides distasonis* ↓ *Faecalibacterium prausnitzii,* and *Bacteroides fragilis* in CD
[37]	*n* = 69; HC *n* = 40; Non-PN SBS *n* = 5; SBS I *n* = 10; SBS II *n* = 14	↑ Lactobacillus * and *Klebsiella,* ↓ *Coprococcus, Faecalibacterium, Lachnospira,* and *Ruminococcus* in SBS patients; ↓ *Blautia, Bacteroides, Odoribacter, Oscillospira, Prevotella, Roseburia,* and *Sutterella* in SBS I and SBS II; ↑ *Streptococcus* and *Staphylococcus* in SBS I
[75]	*n* = 16 NAFLD	↑ *Prevotella copri* and *Prevotella stercorea* in NAFLD
[76]	*n* = 68; HC *n* = 36; NAFLD *n* = 32	↑ *Escherichia coli, Klebsiella pneumoniae,* and *Enterobacter cloacae,* ↓ *Akkermansia muciniphila, Alistipes putredinis, Bacteroides uniformis, Bacteroides fragilis, Oscillibacter* sp., *Ruminococcus bromii, Eubacterium ventriosum,* and *Gemmiger formicilis* in NAFLD
[77]	*n* = 874; Non-NAFLD *n* = 669; NAFLD *n* = 205	↓ *Faecalibacterium* and *Bacteroides* in NAFLD
[78]	*n* = 766; Control *n* = 453; Developed NAFLD *n* = 40; Regressed NAFLD *n* = 35; Persistent NAFLD *n* = 238	↓ *Oscillospira, Odoribacter,* and *Coprococcus* in persistent NAFLD vs. Control; ↓ *Coprococcus eutactus* in regressed NAFLD and persistent NAFLD vs. Control
[79]	*n* = 67; HC *n* = 37; NAFLD *n* = 30	↑ *Porphyromonas, Succinivibrio, Clostridium, Blautia, Dorea, Peptococcus, Mitsuokella,* and *Slackia,* ↓ *Odoribacter, Proteus,* and *Coprococcus* in NAFLD
[80]	*n* = 47; HC *n* = 22; NAFLD *n* = 25	↑ *Escherichia-Shigella*, *Blautia, Clostridium XVIII,* and *Streptococcus,* ↓ *Prevotella* and *Faecalibacterium* in NAFLD
[81]	*n* = 202; no-NAFLD *n* = 31; NAFLD *n* = 171	↑ *Citrobacter,* ↓ *Coprococcus* and *Lachnospira* in significant fibrosis
[82]	*n* = 126; no-NAFLD *n* = 83; NAFLD *n* = 43	↓ Coprococcus, Pseudobutyrivibrio, Moryella, Roseburia, Anaerosporobacter, Anaerotruncus, Ruminococcus, Lactobacillus * in NAFLD
[83]	*n* = 75; HC *n* = 25; NAFLD *n* = 25; NASH *n* = 25	↑ *Bacteroides* and *Prevotella,* ↓ *Faecalibacterium* in NAFLD and NASH
[84]	*n* = 86; Mild/moderate NAFLD *n* = 72; Fibrosis *n* = 14	↑ *Eubacterium rectale* in mild/moderate NAFLD; ↑ *Bacteroides vulgatus* and *Escherichia coli,* ↓ *Ruminococcus obeum,* and *Eubacterium rectale* in fibrosis
[85]	*n* = 24; HC *n* = 8; NASH *n* = 16	↑ *Phascolarctobacterium* in NASH
[86]	*n* = 67; HC *n* = 28; NASH *n* = 24; SS *n* = 15	↓ *Ruminococcus, Faecalibacterium prausnitzii,* and *Coprococcus* in NAFLD and SS vs. HC
[87]	*n* = 50; HC *n* = 17; NASH *n* = 22; SS *n* = 11	↓ *Clostridium coccoides* in NASH
[88]	*n* = 60; Non significant fibrosis *n* = 35; Significant fibrosis *n* = 25	↑ *Bacteroides* and Lactobacillus *, ↓ *Bifidobacterium* in significant fibrosis
[89]	*n* = 40; NT *n* = 15; HT *n* = 25	↑ *Rothia* ↓ *Faecalicoccus, Morganella, Acetohalobium,* and *Phaeodactylibacter* in HT
[90]	*n* = 70; NT *n* = 47; HT *n* = 23	↑ *Acidaminococcus, Eubacterium,* and *Alistipes* in HT
[91]	*n* = 80; NT *n* = 32; HT *n* = 48	↑ *Ligilactobacillus salivarius, Bacteroides plebeius,* and *Eggerthella,* ↓ *Roseburia faecis, Faecalibacterium prausnitzii, Parabacteroides distasonis,* Unclassified *Fusobacterium,* and *Coprobacillus* in HT
[92]	*n* = 120; HC *n* = 60; HT *n* = 60	↑ *Klebsiella, Clostridium, Streptococcus, Parabacteroides, Eggerthella,* and *Salmonella,* ↓ *Faecalibacterium,* and *Roseburia* in HT
[93]	*n* = 196; HC *n* = 41; pHT *n* = 56; HT *n* = 99	↑ *Prevotella* and *Klebsiella* in pHT or HT; ↑ *Porphyromonas* and *Actinomyces* in HT; ↓ *Faecalibacterium, Oscillibacter, Roseburia, Subdoligranulum, Blautia, Bifidobacterium, Coprococcus, Butyrivibrio, Eggerthella, Streptococcus,* and *Akkermansia* in pHT and HT
[94]	*n* = 900; HC *n =* 300; HT *n =* 300; CAD *n* = 300	↑ *Escherichia* in HT
[95]	*n* = 235; HC *n* = 42; NH *n* = 63; AH *n* = 104; HLD *n* = 26	↑ *Blautia, Bacteroides,* and *Faecalibacterium* in NH; ↑ *Bacteroides* and *Faecalibacterium* in HLD and HC
[96]	*n* = 502; HC *n =* 100; ACS *n =* 402	↑ *Escherichia coli* and *Streptococcus,* ↓ Lactobacillus * in ACS
[97]	*n* = 64; HC *n =* 32; CAS *n =* 32	↑ *Acidaminococcus, Christensenella,* and Lactobacillus *, ↓ *Anaerostipes, Fusobacterium, Gemella, Parvimonas, Romboutsia,* and *Clostridium XVIII/XlVa/XlVb* in CAS
[98]	*n* = 345; No SCA *n* = 201; SCA *n* = 144	↑ *Escherichia* and *Oscillospira* in SCA
[99]	Sweden cohort *n* = 25; Control 1 *n* = 13; Atherosclerosis 1 *n* = 12; China cohort *n* = 385; Control 2 *n* = 171; Atherosclerosis 2 *n* = 214	↑ *Bifidobacterium adolescentis, Collinsella aerofaciens, Blautia hydrogenotrophica,* and *Anaerotruncus colihominis* in atherosclerosis 1; ↑ *Bacteroides fragilis, Streptococcus salivarius, Clostridium nexile, Ruminococcus gnavus, Ruminococcus torques, coli, Klebsiella pneumoniae,* and *Akkermansia muciniphila* in atherosclerosis 2
[100]	*n* = 106; Control *n* = 53; CAD *n* = 53	↑ *Porphyromonas, Prevotella, Agathobacter, Ruminococcus gnavus, Catenibacterium,* and *Succiniclasticum,* ↓ *Anaerosporobacter, Coprococcus, Eisenbergiella, Fusocatenibacter, Eubacterium hallii, Ruminococcus gauvreauii, Fournierella,* and *Veillonella* in CAD
[101]	*n* = 201; HC *n* = 40; CAD *n* = 161	↑ *Actinomyces, Haemophilus, Granulicatella, Weissella, Veillonella, Streptococcus, Klebsiella, Rothia, Enterococcus* (CAG17); ↓ *Faecalibacterium, Roseburia, Oscilibacter* (CAG4); *Ruminococcus 2, Dorea, Blautia, Clostridium XVIII* (CAG14); Anaerostipes, Blautia, Lactobacillus *, Fusocatenibacter, Clostridium XIVa, Gemella, Bifidobacterium, Saccharibacteria genera incertae sedis (CAG15); *Roseburia, Clostridium XIVb, Parasutterella, Butyricicoccus* (CAG16) in CAD
[102]	*n* = 405; HC *n* = 187; ACVD *n* = 218	*↑ Escherichia coli, Klebsiella* spp., *Enterobacter aerogenes, Streptococcus* spp., *Ligilactobacillus salivarius, Solobacterium moorei, Atopobium parvulum, Ruminococcus gnavus,* and *Eggerthella lenta, ↓ Roseburia intestinalis, Faecalibacterium prausnitzii, Bacteroides* spp., *Prevotella copri,* and *Alistipes shahii* in ACVD

AbOB: abdominal obesity; ACS: acute coronary syndrome; ACVD: atherosclerotic cardiovascular disease; AH: hypertensive patients undergoing anti-hypertensive treatment; CAD: coronary artery disease; CAG: co-abundance group; CAS: carotid atherosclerosis; CD: Crohn’s disease; CN: cognitive normal group; CRC: colorectal cancer; DD: diverticular disease; DR: diabetic retinopathy; Dys: dyslipidemia; DZ: dizygotic twin pairs; HC: healthy control; HL: hyperlipidemia; HLD: normal blood pressure but with hyperlipidemia; HT: hypertension; IBD: inflammatory bowel disease; IBS: irritable bowel syndrome; IFG: impaired fasting glycemia; IGT: impaired glucose tolerance; KnownT2D: diabetics on antidiabetic treatment; LN: lean; Mets: metabolic syndrome; MODY2: maturity-onset diabetes of the young 2; MZ: monozygotic twin pairs; NAFLD: non-alcoholic fatty liver disease; NASH: non-alcoholic steatohepatitis; NewT2D: newly diagnosed diabetic; NGT: normal glucose tolerance; NH: hypertensive patients with treatment-naive hypertension; Non-PN SBS: parenteral nutrition-independent short bowel syndrome; NT: normotension; OB: obese; OW: overweight; pHT: prehypertension; PSC: primary sclerosing cholangitis; pT2D: prediabetic; RISK1: patients with only one disease; RISK2: patients with two diseases; RISK3: patients with three diseases; SBS I: parenteral nutrition-dependent short bowel syndrome I; SBS II: parenteral nutrition-dependent short bowel syndrome II; SCA: subclinical carotid atherosclerosis; SS: simple steatosis; T1D: type 1 diabetes; T2D: type 2 diabetes; T2D+: type 2 diabetes with chronic complications; T2D-: type 2 diabetes without chronic complications; T2DCI: type 2 diabetes cognitive impairment group; UC: ulcerative colitis. * *Lactobacillus* includes species from Lactobacillaceae family [22]. ↑ Taxa increasement and ↓ Taxa decreasement.

**Table 2:** Disease/health-related metabolites and chemical classification.

Health-Related Metabolites	Disease-Related Metabolites
**Fatty Acid Pathways—Metabolites and conjugates**	
10-Heptadecenoate (17:1n7)	(+)-Cucurbic acid
2-Hydroxyhexadecanoate	12,13-Dihydroxy-11-methoxy-9-octadecenoic acid
Acetate	17-Oxo-octadecanoic acid
Azelaic acid	2-Hydroxyadipate
Caproic acid	2-Methyl-tridecanedioic acid
Caprylic acid	3-Keto stearic acid
Isovalerate	8,11,14-Eicosatrienoic acid
Undecanedionate	8Z-Decen-4,6-diynoic acid
	9,10-Dichloro-octadecanoic acid
	Adrenic acid
	Arachidonic acid
	Diamino-pimelic acid
	Dihomo-linolenate (20:3n3 or n6)
	Docosahexaenoic acid
	Docosanedioic acid
	Eicosatrienoic acid
	Linolenic acid
**Amino Acid Pathways—Metabolites and derivatives**	
Glycylvaline	Asymmetric dimethylarginine (ADMA)
Isoleucine	Carnosine
N6,N6,N6-Trimethyllysine	Cinnamoylglycine
N-Acetylalanine	Citrulline
S-Carboxymethyl-L-cysteine	Ɣ-Glutamylglutamine
Valine	Glycine
	Homocitrulline
	Homocysteine
	L-Lysine
	N6-Carboxymethyllysine
	Nɑ-Acetyl-L-arginine
	Propionylglutamine
**Biliary Acid Pathways—Metabolites and derivatives**	
Chenodeoxyglycocholate	12-Dehydrocholic acid
Glycoursodeoxycholic acid	3-Dehydrocholic acid
	3β-Cholic acid
	6,7-Diketolithocholic acid
	6-Keto-Lithocholic acid
	7,12-Diketolithocholic acid
	7-Dehydrocholic acid
	7-Ketolithocholic acid Allocholic acid
	Chenodeoxycholic acid
	Chenodeoxycholic acid-3Gln Cholate sulfate
	Dehydrocholic acid
	Glycochenodeoxycholic acid
	Glycodeoxycholic acid
	Glycolithocholic acid
	Hyodeoxycholic acid
	Lithocholic acid
	Murocholic acid
	Nordeoxycholic acid
	Taurocholic acid
	Taurohyocholic acid
	Taurolithocholic acid
	Tauroursodeoxycholic acid
	αMuricholic acid
	βDeoxycholic acid
	βMuricholic acid

## Data Availability

Not applicable.

## Supplementary Materials

The following supporting information can be downloaded at: https://www.mdpi.com/article/10.3390/ijms24054519/s1.
