# Supplementary material for: Key Stratification of Microbiota Taxa and Metabolites in the Host Metabolic Health–Disease Balance
Source: Int J Mol Sci. 2023 Feb 24;24(5):4519. doi: 10.3390/ijms24054519 (PMC10003303; doi:10.3390/ijms24054519)
Supplement: Supplementary file 1 [file ijms-24-04519-s001.zip › Supplementary material 2.pdf]

Supplementary material S2. Microbiota metabolite modifications and microbiota variation in several pathologies. *Gut microbiota-metabolite correlation +/-

Ref., Sample size, Biosample	Gut microbiota taxa modification	Metabolite modifications	*	Health status
[103] Northern Finland Birth Cohort *n* = 12,058; microbiome *n* = 563; metabolome *n* = 340 Faeces	*Blautia*	N-Acetilalanine, Ursodeoxycholate, N6,N6,N6-Trimethyllysine, 7-Methylguanine, Isoursodeoxycholate, Deoxycholate, N-Propionylalanine	+	NW
	Ruminococcus	Cholate sulfate, Dihomo-linolenate (20:3n3 or n6), 5α-Androstan-3α,17α-diol disulfate, Homocitrulline, Androsterone sulfate, Palmitoylcarnitine (C16), Propionylglutamine, Isoursodeoxycholate, 5α-Androstan-3α,17β-diol monosulfate, Deoxycholate, 2-Acetamidobutanoate, Phytosphingosine, N-Propionylalanine, Oleoylcarnitine (C18:1), Oleoyl ethanolamide, Glycodeoxycholate, N6-Carboxymethyllysine	+	OW
	Blautia	Bilirubin (E,Z or Z,E), I-Urobilinogen, D-Urobilin, Stearoyl ethanolamide, N1-Methyl-2-pyridone-5-carboxamide, 7-Methylguanine, Carnosine, 2-Hydroxyadipate, Thymidine	+	OW
	Roseburia	Nicotinate, Pantothenate	+	OW
	Oscillospira	Cholate sulfate	+	OW
	Eggerthella	5α-Androstan-3β,17β-diol monosulfate, Pregnenolone steroid monosulfate, Pregnenolone sulfate	+	OW
	Haemophilus	Ribonate	+	OW
	Veillonella	Arabonate/xylonate	+	OW
[104] *n* = 33; OB/NoPCOS *n* = 15; OB/PCOS *n* = 18 Faeces	*Parabacteroides*	Teasterone	+	OB/PCOS
	Veillonella	Arachidonic acid, 8,11,14-Eicosatrienoic acid, Docosahexaenoic acid, Adrenic acid	+	
		Taurocholic acid	-	
	Lachnospira	Arachidonic acid, 8,11,14-Eicosatrienoic acid, Docosahexaenoic acid, Adrenic acid	+	
		Taurocholic acid	-	
	Fusobacterium	Teasterone	-	
	Lachnoclostridium	Teasterone	-	
	Eubacterium coprostanoligenes group	Teasterone	+	

	Ruminococcaceae UCG-002	Teasterone	+	
	Erysipelotrichaceae UCG-003	Dehydroepiandrosterone-sulfate	-	
	Lachnospiraceae NK4A136 group	Teasterone	+	
	norank *Muribaculaceae*	Dehydroepiandrosterone-sulfate	+	
[105]	*Akkermansia*	Indolepropionate	+	LH
n = 1280; LH *n* =	*Barnesiella*	Valine	+	LH
633; ObH *n* = 494;	*Clostidium IV*	Tyrosine	+	LH
ObT2D *n* = 153		Isovalerate, Glutamine, Oxalate	-	LH
n = 400; LH *n* =	*Clostridium XlVa*	Phenylalanine, Glycylvaline	+	LH
228; ObH *n* = 145;		Glutamine, 3-Phenylpropionate, Inosine	-	LH
ObT2D *n* = 27	*Prevotella*	3-Phenylpropionate	+	LH
subset	*Intestimonas*	N1-Methyl-2-pyridone-5-carboxamide	+	LH
Serum	*Barnesiella*	Docosapentaenoate	-	LH
	Butyricicoccus	1-Stearoyl-GPE (18:0)	+	LH
	Anaerovorax	2-Methylbutyrylcarnitine	+	LH
		Adrenate (22:4n6), Epiandrosterone sulfate	-	ObH
	Parabacteroides	Indolepropionate, 1-Linoleoyl-GPC (18:2), 1-Dihomo-linoleoyl-GPC (20:2)	+	ObH
		10-Heptadecenoate (17:1n7)	-	LH
	Ruminococcus	Gamma-CEHC	+	ObH
		1-Arachidonoyl-GPI (20:4)	-	LH
	Odoribacter	Indolepropionate, 3-Phenylpropionate	+	ObH
		Glycylvaline	-	LH
	Butyricimonas	3-Phenylpropionate, 2-Linoleoyl-GPC (18:2), 1-Linoleoyl-GPC (18:2)	+	ObH
	Oscillibacter	Citrulline, 1-Oleoyl-GPC (18:1), Cinnamoylglycine	+	ObH
	Anaerotruncus	1-Linoleoyl-GPC (18:2)	+	ObH
[106]	*Fusobacterium varium*	Leucine, Glutamate	+	OB MLGs
n = 151; LC *n* = 79;	*Fusobacterium ulcerans*	Leucine, Glutamate	+	OB MLGs
OB *n* = 72	*Ruminococcus torques*	Glutamate, Phenylalanine	+	OB MLGs
	Dorea longicatena	Phenylalanine, Glutamate	+	OB MLGs

Serum		Glutamine	-	OB MLGs
	Eubacterium hallii	Glutamate	+	OB MLGs
	Ruminococcus spp.	Glutamate	+	OB MLGs
		Glutamine	-	OB MLGs
	Coprococcus comes	Glutamate	+	OB MLGs
		Glutamine	-	OB MLGs
	Lachnospiraceae bacterium	Leucine, Glutamate, Phenylalanine	+	OB MLGs
	Bacteroides spp.	Valine, Leucine, Isoleucine, Phenylalanine, Tyrosine, Glutamate	-	Control MLGs
	Veillonella spp.	Glutamate	-	Control MLGs
	Haemophilus parainfluenzae	Glutamate	-	Control MLGs
	Bacteroides thetaiotaomicron	Valine, Leucine, Phenylalanine, Tyrosine, Glutamate	-	Control MLGs
	Bacteroides ovatus	Valine, Phenylalanine, Tyrosine, Glutamate	-	Control MLGs
	Bacteroides intestinalis	Valine, Leucine, Isoleucine, Phenylalanine, Tyrosine, Glutamate	-	Control MLGs
	Holdemania filiformis	Valine, Leucine	-	Control MLGs
	Faecalibacterium prausnitzii	Valine, Leucine, Isoleucine, Phenylalanine, Glutamate	-	Control MLGs
	Clostridiales bacterium	Valine, Isoleucine, Phenylalanine, Tyrosine, Glutamate	-	Control MLGs
	Anaerotruncus colihominis	Valine, Leucine, Isoleucine, Phenylalanine	-	Control MLGs
	Bacteroides uniformis	Valine, Leucine, Phenylalanine, Tyrosine	-	Control MLGs
	Klebsiella pneumoniae	Glutamate	-	Control MLGs
	Dialister invisus	Valine, Phenylalanine, Glutamate	-	Control MLGs
	Alistipes spp.	Tyrosine	-	Control MLGs
	Alistipes putredinis	Valine, Leucine	-	Control MLGs
	Bacteroides xylanisolvens	Valine	-	Control MLGs
	Akkermansia muciniphila	Phenylalanine	-	Control MLGs
	Odoribacter splanchnicus	Phenylalanine	-	Control MLGs
	Alistipes shahii	Tyrosine	-	Control MLGs
[107]	Enterococcaceae	Octadecenoic acid (Diet), N-Acetyl-DOPA(Gut-Brain)	+	ND, ↓ OB
n = 16; NW *n* = 8;	Enterobacteriaceae	Octadecenoic acid (Diet)	+	ND
OB *n* = 8		Ascorbic acid (Diet)	-	ND
Faeces	Christensenellaceae	Azelaic acid (Diet), Suberic acid (Diet)	+	ND, ↓ OB

	Succinivibrionaceae	Suberic acid (Diet)	+	↓ OB
	Coriobacteriaceae	Deoxycholic acid (Liver)	+	↑ OB
	Propionibacteriaceae	Urobilin (Liver), Deoxycholic acid (Liver)	+	ND, ↑ OB
	Synergistaceae	Deoxycholic acid (Liver)	+	↑ OB
	Erysipelotrichaceae	Dimethyl-2-oxoglutarate (Gut-Endogen)	+	↓ OB
		Ascorbic acid (Diet)	-	ND
	Ruminococcaceae	Dimethyl-2-oxoglutarate (Gut-Endogen)	+	↓ OB
		Tripeptide (Diet)	-	↑ OB
	Prevotellaceae	Ascorbic acid (Diet)	+	ND
	Clostridiaceae	Hydroxyhexadecanedioic acid (Diet)	+	ND
	Rhodobacteraceae	Hydroxyhexadecanedioic acid (Diet), Azelaic acid (Diet)	+	ND, ND
	Desulfovibrionaceae	Hydroxyhexadecanedioic acid (Diet), Azelaic acid (Diet)	+	ND, ND
	Streptococcaceae	N-Acetyl-DOPA (Gut-Brain)	-	↓ OB
	Porphyromonadaceae	Hydroxyphenyl propionic acid (Diet), Tripeptide (Diet), 12-ketodeoxycholic acid (Liver)	+	ND, ↑ OB, ↑ OB
	Defluviitaleaceae	Hydroxyphenyl propionic acid (Diet), Tripeptide (Diet), 12-ketodeoxycholic acid (Liver)	+	ND, ↑ OB, ↑ OB
	Staphylococcaceae	Homovanillic acid (Gut-Brain)	+	ND
	Alcaligenaceae	Rosmarinic acid (Diet), Quinic acid (Diet), Homovanillic acid (Gut-Brain)	+	ND, ND, ND
	Helicobacteraceae	Homovanillic acid (Gut-Brain)	+	ND
	Bacteroidaceae	Quinic acid (Diet), Tripeptide (Diet), Homovanillic acid (Gut-Brain)	+	ND, ↑ OB, ND
		Dimethyl-2-oxoglutarate (Gut-Endogen), Suberic acid (Diet), Urobilin (Liver)	-	↓ OB, ↓ OB, ND
	U. m. of Clostridiales order	Dimethyl-2-oxoglutarate (Gut-Endogen), N-Acetyl-DOPA (Gut-Brain)	+	↓ OB, ↓ OB
	U. m. of Bacteroidales order	Hydroxyphenyl propionic acid (Diet), Tripeptide (Diet), 12-ketodeoxycholic acid (Liver)	+	ND, ↑ OB, ↑ OB
	Tenericutes	Suberic acid (Diet)	+	↓ OB
	Firmicutes	Dimethyl-2-oxoglutarate (Gut-Endogen)	+	↓ OB

		Quinic acid (Diet), Tripeptide (Diet), Urobilin (Liver)	-	ND, ↑ OB, ND
	Bacteroidetes	Tripeptide (Diet)	+	↑ OB
		Dimethyl-2-oxoglutarate (Gut-Endogen), Suberic acid (Diet)	-	↓ OB, ↓ OB
[108]<br>*n* = 100; HC *n* = 35; T2D+ *n* = 49; T2D- *n* = 16<br>Faeces	*Enhydrobacter*<br>*Acinetobacter*<br>*Pseudomonas*<br>*Aeromonas*<br>*Providencia*	LDL cholesterol	+	T2D+
	M5<br>*Akkermansia*	HDL cholesterol	+	HC
	MM12<br>*Sarcina*<br>Mollicutes<br>Tenericutes	Cholic acid	-	HC
	MM13<br>*Solibacillus*<br>*Oligella*<br>*Epulopiscium*<br>*Parapusillimonas*<br>*Psychrobacter*<br>*Flavobacterium*	Acetate, Glycoursodeoxycholic acid, Chenodeoxyglycocholate	-	HC
	MM14<br>*Shuttleworthia*<br>*Peptoniphilus*<br>*Atopobium*<br>*Anaerococcus*	Palmitoylcarnitine, Diacylglycerol (15:0/18:3), Diacylglycerol (15:0/20:3)	+	T2D+
	MM15<br>*Allisonella*<br>*Ochrobactrum*<br>*Butyricicoccus*<br>*Anaerostipes*	Lysophosphatidylcholine (18:2), Phosphatidylcholine (16:0/17:0)	+	T2D+

	Ruminococcus toques group			
	MM16			
	Veillonella	Linolenic acid, Lysophosphatidylcholine (18:2)	+	T2D+
	Weissella			
	Pseudobutyrivibrio			
	Streptococcus	Butyrate	-	
	Prevotellaceae UCG-003			
	MM19			
	Blautia	HDL cholesterol	-	T2D-
	Marvinbryantia			
	Lachnospiraceae NK4A136 group			
	MM32			
	Morganella	Acetate, Butyrate, Glycocholic acid, Cholic acid	-	HC
	Campylobacter			
	MM34			
	Lactococcus	Palmitoylcarnitine, Lysophosphatidylcholine (18:2), Phosphatidylcholine (16:0/17:0)	+	T2D+
	Prevotella			
	MM35			
	Eubacterium oxidoreducens group	Glycoursodeoxycholic acid, Chenodeoxyglycocholate, Glycocholic acid	-	HC
	Prevotellaceae NK3B31 group			
	MM38			
	Neisseria	Cholic acid	+	T2D+
	MM40			
[109] *n* = 60; NGT *n* = 20; T2D *n* = 20; IGR *n* = 20 Faeces	*Blautia*	PA (O-16:0/12:0), 24-Nor-9,11-seco-11-acetoxy-3,6-dihydroxycholest-7,22-dien-9-one, Matricin, 8Z-Decen-4,6-diynoic acid, Docosanedioic acid, Methyl, Ikarisoside	-	T2D vs. IGR
	Coprococcus 3	Matricin, (6S)-Dehydrovomifoliol	-	T2D vs. IGR
	Lactobacillaceae	14,15-HxA3-D (11S), Cytochalasin, Tanariflavanone	-	T2D vs. IGR
	Subdoligranulum	PA (O-16:0/12:0), 24-Nor-9,11-seco-11-acetoxy-3,6-dihydroxycholest-7,22-dien-9-one, Tanariflavanone	-	T2D vs. IGR

Prebotella 9	PA (O-16:0/12:0), 24-Nor-9,11-seco-11-acetoxy-3,6-dihydroxycholest-7,22-dien-9-one	+	T2D vs. IGR
Bacteroides	PI (O-20:0/18:0), 3Z,6Z,9Z,12Z,15Z,19Z,22Z,25Z,28Z-Hentriacontanonaene, 10Z-Pentacosene, SM (d18:1/25:0), TG (13:0/17:1(9Z)/17:1(9Z))[iso3], GlcCer (d18:1/20:0), SM (d18:1/24:0), GlcCer (d18:2/23:0), TG (15:0/15:0/15:0), 1α,25-Dihydroxy-22-oxavitamin, 3-O-(β-D-glucopyranosyl-(1→6)-β-D-glucopytanosyl), Diamino-pimelic acid	+	T2D vs. IGR
Fusicatenibater	24-Nor-9,11-seco-11-acetoxy-3,6-dihydroxycholest-7,22-dien-9-one	-	T2D vs. IGR
Faecalibacterium	17-Oxo-octadecanoic	+	T2D vs. IGR
	PA (O-16:0/12:0), 24-Nor-9,11-seco-11-acetoxy-3,6-dihydroxycholest-7,22-dien-9-one, PC (O-12:0/O-2:0)	-	T2D vs. IGR
Dorea	26,26,26,27,27,27-Hexafluoro-25-hydroxy-23,23,24,24-tetradehydrovitamin	-	T2D vs. IGR
Rummicococcus torques group	LysoPC (15:0), 17-Oxo-octadecanoic	+	T2D vs. IGR
Lachnospiraceae NK4A136 group	(3S)-3′,7-Dihydroxy-2′,4′,5′,8-tetramethoxyisoflavan	+	T2D vs. IGR
	1α,25-Dihydroxy-22-oxavitamin	-	T2D vs. IGR
Butyricicoccus	PI (O-20:0/18:0), 3Z,6Z,9Z,12Z,15Z,19Z,22Z,25Z,28Z-Hentriacontanonaene, GalNAcβ1-4Galβ1-4Glcβ-Cer(d18:1/24:1(15Z)), TG (15:0/15:0/15:0), SM(d18:1/24:0), PG (O-20:0/21:0), 10Z-Pentacosene, 21-Methyl-8Z-pentatriacontene, (6S)-Dehydrovomifoliol	+	IGR vs. NGT
Bacteroides	Ɣ-Glutamylglutamine, L-Lysine, MGDG (20:5(5Z,8Z,11Z,14Z,17Z)/18:3(9Z,12Z,15Z))	+	IGR vs. NGT
Lachnoclostridium	L-Lysine	+	IGR vs. NGT
Roseburia	1α,25-Dihydroxy-22-oxavitamin	+	IGR vs. NGT
Faecalibacterium	L-Tyrosine, DL-2-hydroxy, 21-Methyl-8Z-pentatriacontene	+	IGR vs. NGT
Blautia	5a-Cholestane-3a,7a,12a,23,25-pentol	+	IGR vs. NGT
Coprococcus 1	L-Lysine, Scillaren	+	IGR vs. NGT
Fusicatenibacter	Ɣ-Glutamylglutamine, L-Tyrosine, L-Lysine	+	IGR vs. NGT

Subdoligranulum	7-Dehydro-desmosterol, 24-Keto-25dehydrocholesterol, Simvastatin, 5a-Cholestane-3a,7a,12a,23,25-pentol, (22S)-1α,22,25-Trihydroxy-23,24-tetradehydro-24a-homo-20-epivitamin, PA (14:1/(9Z)/0:0), Manoalide, PA (17:0/21:0), Teasterone, Hippuristanolide	+	IGR vs. NGT
Collinsella	Afrormosin, PG (P-16:0/15:0)	+	IGR vs. NGT
Ruminococcus torques group	2-Methyl-tridecanedioic, 27-Nor-campestan-3β,4β,5α,6α,7β,8β,14α,15α,24-nonol, LysoPC (14:1(9Z))	+	IGR vs. NGT
Eubacterium hallii group	L-Tyrosine, L-Lysine	+	IGR vs. NGT
Eubacterium rectale group	L-Lysine, Dodecanedioic, (+)-Cucurbic acid	+	IGR vs. NGT
Eubacterium coprostanoligenes group	7-Dehydro-desmosterol, 24-Keto-25dehydrocholesterol, Simvastatin, 5α-Cholestane-3α,7α,16α,26-tetrol, 5a-Cholestane-3a,7a,12a,23,25-pentol, Scillaren, PA (14:1/(9Z)/0:0), Manoalide, PA (17:0/21:0), Hippuristanolide	+	IGR vs. NGT
Lachnospiraceae UCG-004	1α,25-Dihydroxy-22-oxavitamin	+	IGR vs. NGT
Lachnospiraceae NC2004 group	L-Lysine, DL-2-hydroxy, Enkephalin, Ptilosteroid, 7E,9E,11-Dodecatrienyl, Hexacosanoic, Hexacosanedioic, Artonin, Viscutin	+	IGR vs. NGT
Lachnospiraceae ND3007 group	L-Lysine	+	IGR vs. NGT
Lachnospiraceae NK4A136 group	L-Tyrosine, 7-Dehydro-desmosterol, 24-Keto-25dehydrocholesterol, Simvastatin, 5α-Cholestane-3α,7α,16α,26-tetrol, 5a-Cholestane-3a,7a,12a,23,25-pentol, Scillaren	+	IGR vs. NGT
Bifidobacterium	Cristacarpin	+	T2D vs. NGT
Bacteroides	12,13-Dihydroxy-11-methoxy-9-octadecenoic acid, Cer (d18:0/h 17:0)	+	T2D vs. NGT
Parabacteroides	12,13-Dihydroxy-11-methoxy-9-octadecenoic acid, Cer (d18:0/h 17:0)	+	T2D vs. NGT
Intestinibacter	12,13-Dihydroxy-11-methoxy-9-octadecenoic acid	+	T2D vs. NGT
Subdoligranulum	12,13-Dihydroxy-11-methoxy-9-octadecenoic acid, Cer (d18:0/h 17:0)	+	T2D vs. NGT
Fusicatenibacter	12,13-Dihydroxy-11-methoxy-9-octadecenoic acid	+	T2D vs. NGT
Ruminococcaceae UCG-005	Piceid, ɣ-Glutamylglutamine	+	T2D vs. NGT

	Lachnospiraceae NK4A136 group	Cristacarpin, 24-Keto-25dehydrocholesterol, 5a-Cholestane-3a,7a,12a,23,25-pentol	+	T2D vs. NGT
[110] n = 9180; n = 759 HCHS/SOL participants Serum	*Ruminococcus, Flavonifractor, Faecalitalea, Lachnoclostridium, Faecalibacterium, Subdoligranulum, Clostridium, Lachnoanaerobaculum, Intestinimonas, Erysipelatoclostridium, Cellulomonas, Eubacterium, Fournierella, Dorea, Butyrivibrio, Eggerthella, Pseudoflavonifractor, Bifidobacterium, Porphyromonas, Parabacteroides, Bittarella,*	Indolepropionate	+, -, +, -, +, +, +, +, +, -, +, +, +, +, +, -, +, +, +, -, +	↓ T2D
	Cellulomonas, Fournierella, Methanobrevibacter, Anaerostipes, Catenibacterium	Indoleacetate	ND	ND
	Faecalitalea, Pseudoflavonifractor, Parabacteroides, Alistipes, Akkermansia, Faecalicatena, Intestinibacter, Odoribacter, Gordonibacter, Sanguibacteroides, Anaerotruncus	Indoxyl sulfate	ND	ND
[111] n = 69; HC n = 40; Non-PN SBS n = 5; SBS I n = 10; SBS II n = 14 Faeces and serum	Pseudomonadaceae	2-Pentyl furan, Nonanal, Dodecanoic acid, Decanoic acid, Chenodeoxycholic acid, Deoxycholic acid	+	SBS
		Pentanoic acid, Butanoic acid, Lithocholic acid	-	HC
	Staphylococcaceae	2-Pentyl furan, Hexanal, Octanal, Decanoic acid, Taurocholic acid, Chenodeoxycholic acid, Deoxycholic acid	+	SBS
		Ɣ-Undecalactone, Geranil acetone, Butanoic acid	-	HC
	Enterococcaceae	2-Pentyl furan, Hexanal, Octanal, Nonanal, Decanoic acid, Chenodeoxycholic acid, Deoxycholic acid	+	SBS
		Indole, ɣ-Undecalactone, Geranil acetone, Pentanoic acid, Butanoic acid, Lithocholic acid	-	HC
	Fusobacteriaceae	2-Pentyl furan, Hexanal, Octanal, Nonanal, Taurocholic acid, Chenodeoxycholic acid, Deoxycholic acid	+	SBS

		Ɣ-Undecalactone, Geranil acetone, Pentanoic acid, Butanoic acid	-	HC
	Enterobacteriaceae	2-Pentyl furan, Hexanal, Octanal, Nonanal, Dodecanoic acid, Decanoic acid, Taurocholic acid, Chenodeoxycholic acid, Deoxycholic acid, Cholic acid, Glycocholic acid, Glycochenodeoxycholic acid	+	SBS
		Ɣ-Undecalactone, p-Cresol, 1-Nonanol, Pentanoic acid, Butanoic acid, Lithocholic acid	-	HC
	Lactobacillaceae	2-Pentyl furan, Hexanal, Octanal, Nonanal, Dodecanoic acid, Decanoic acid, Taurocholic acid, Chenodeoxycholic acid, Deoxycholic acid, Cholic acid	+	SBS
		Indole, ɣ-Undecalactone, Geranil acetone, p-Cresol, 1-Nonanol, Pentanoic acid, Butanoic acid, Lithocholic acid	-	HC
	Streptococcaceae	2-Pentyl furan, Hexanal, Octanal, Nonanal, Dodecanoic acid, Decanoic acid, Taurocholic acid, Chenodeoxycholic acid, Deoxycholic acid, Glycochenodeoxycholic acid	+	SBS
		Indole, ɣ-Undecalactone, Geranil acetone, p-Cresol, 1-Nonanol, Pentanoic acid, Butanoic acid, Lithocholic acid	-	HC
	Clostridiaceae	Butanoic acid, 1-Nonanol	+	HC
		2-Pentyl furan, Hexanal, Octanal, Nonanal, Dodecanoic acid, Decanoic acid, Chenodeoxycholic acid, Deoxycholic acid, Glycodeoxycholic acid	-	SBS
	Coriobacteriaceae	Pentanoic acid, p-Cresol, Lithocholic acid	+	HC
		2-Pentyl furan, Hexanal, Octanal, Nonanal, Dodecanoic acid, Phenol, Taurocholic acid, Chenodeoxycholic acid, Deoxycholic acid	-	SBS
	Rikenellaceae	Butanoic acid, Pentanoic acid, 1-Nonanol, p-Cresol, Geranil acetone, ɣ-Undecalactone, Indole, Lithocholic acid	+	HC
		2-Pentyl furan, Hexanal, Octanal, Nonanal, Dodecanoic acid, Decanoic acid, Phenol, Taurocholic acid, Chenodeoxycholic acid, Deoxycholic acid, Glycochenodeoxycholic acid	-	SBS

Ruminococcaceae	Butanoic acid, Pentanoic acid, 1-Nonanol, p-Cresol, Geranil acetone, ɣ-Undecalactone, Lithocholic acid	+	HC
	2-Pentyl furan, Hexanal, Octanal, Nonanal, Dodecanoic acid, Decanoic acid, Taurocholic acid, Chenodeoxycholic acid, Deoxycholic acid, Cholic acid, Glycocholic acid, Glycochenodeoxycholic acid	-	SBS
Barnesiellaceae	Butanoic acid, Pentanoic acid, 1-Nonanol, p-Cresol, Geranil acetone, ɣ-Undecalactone, Indole, Lithocholic acid	+	HC
	2-Pentyl furan, Hexanal, Octanal, Nonanal, Dodecanoic acid, Decanoic acid, Taurocholic acid, Chenodeoxycholic acid, Deoxycholic acid, Cholic acid, Glycocholic acid, Glycochenodeoxycholic acid	-	SBS
Odoribacteraceae	Butanoic acid, Pentanoic acid, 1-Nonanol, p-Cresol, Geranil acetone, ɣ-Undecalactone, Lithocholic acid	+	HC
	2-Pentyl furan, Hexanal, Octanal, Nonanal, Dodecanoic acid, Decanoic acid, Taurocholic acid, Chenodeoxycholic acid, Deoxycholic acid, Cholic acid, Glycochenodeoxycholic acid	-	SBS
Porphyromonadaceae	Butanoic acid, Pentanoic acid, 1-Nonanol, p-Cresol, Lithocholic acid	+	HC
	2-Pentyl furan, Hexanal, Octanal, Nonanal, Dodecanoic acid, Decanoic acid, Chenodeoxycholic acid, Deoxycholic acid	-	SBS
Paraprevotellaceae	Butanoic acid, Pentanoic acid, 1-Nonanol, p-Cresol, ɣ-Undecalactone, Lithocholic acid	+	HC
	2-Pentyl furan, Hexanal, Octanal, Nonanal, Dodecanoic acid, Decanoic acid, Taurocholic acid, Chenodeoxycholic acid, Deoxycholic acid	-	SBS
Prevotellaceae	Butanoic acid, Pentanoic acid, 1-Nonanol, p-Cresol, Geranil acetone, Lithocholic acid	+	HC
	2-Pentyl furan, Hexanal, Octanal, Nonanal, Dodecanoic acid, Decanoic acid, Chenodeoxycholic acid, Deoxycholic acid, Glycochenodeoxycholic acid	-	SBS

	S24 7	Butanoic acid, p-Cresol, Geranil acetone, Lithocholic acid	+	HC
		2-Pentyl furan, Hexanal, Octanal, Nonanal, Dodecanoic acid, Decanoic acid, Chenodeoxycholic acid, Deoxycholic acid, Cholic acid	-	SBS
	Alcaligenaceae	Butanoic acid, Pentanoic acid, 1-Nonanol, p-Cresol, ɣ-Undecalactone, Indole	+	HC
		Dodecanoic acid, Decanoic acid, Taurocholic acid, Chenodeoxycholic acid, Glycocholic acid	-	SBS
	Bacteroidaceae	Butanoic acid, Pentanoic acid, 1-Nonanol, p-Cresol, ɣ-Undecalactone, Indole, Lithocholic acid	+	HC
		2-Pentyl furan, Hexanal, Octanal, Nonanal, Dodecanoic acid, Decanoic acid, Taurocholic acid, Chenodeoxycholic acid, Deoxycholic acid, Glycocholic acid, Glycochenodeoxycholic acid	-	SBS
	Lachnospiraceae	Butanoic acid, Pentanoic acid, 1-Nonanol, p-Cresol, Geranil acetone, ɣ-Undecalactone, Indole, Lithocholic acid	+	HC
		2-Pentyl furan, Hexanal, Octanal, Nonanal, Dodecanoic acid, Decanoic acid, Taurocholic acid, Chenodeoxycholic acid, Deoxycholic acid, Glycocholic acid, Glycochenodeoxycholic acid	-	SBS
	Clostridiales	Butanoic acid, Pentanoic acid, 1-Nonanol, p-Cresol, Geranil acetone, ɣ-Undecalactone, Lithocholic acid	+	HC
		2-Pentyl furan, Hexanal, Octanal, Nonanal, Dodecanoic acid, Decanoic acid, Phenol, Taurocholic acid, Chenodeoxycholic acid, Deoxycholic acid, Glycochenodeoxycholic acid	-	SBS
[112] *n* = 155; Non-IBD n=34; CD *n* = 68; UC *n* = 53 Faeces	*Eubacterium ventriosum*	Urobilin, 4-Methylcatechol	+	non-IBD
		Eicosatrienoic acid	-	IBD:CD
	Coprococcus catus	Urobilin	+	Non-IBD
		Eicosatrienoic acid	-	IBD:CD
	Roseburia hominis	Urobilin, Dodecanedioic acid	+	Non -IBD
		Eicosatrienoic acid	-	IBD:CD
	Dorea longicatena	Urobilin	+	Non -IBD
	Eubacterium hallii	Urobilin, 3-Methyladipate-pimelate	+	Non -IBD

	Linoleoyl ethanolamide, Docosapentaenoic acid, Eicosatrienoic acid	-	IBD:CD
	C14 carnitine, C3-DC-CH3 carnitine	-	IBD:CD+UC
Eubacterium siraeum	Dodecanedioic acid	+	Non -IBD
	N-Acetylputrescine, Phytosphingosine	-	IBD:CD
Alistipes shahii	Urobilin	+	Non -IBD
	C3-DC-CH3 carnitine	-	IBD:CD+UC
Alistipes putredinis	Urobilin	+	Non -IBD
	Taurine	-	IBD:CD
Alistipes finegoldii	5α-Cholestanol	+	Non -IBD
	Cholate, Chenodeoxycholate	-	IBD:CD
Roseburia inulinivorans	Urobilin, 4-Methylcatechol, Cholestenone	+	Non -IBD
	2-Hydroxyphenethylamine, Linoleoyl ethanolamide, Eicosatrienoic acid	-	IBD:CD
Roseburia intestinalis	Urobilin, Hydrocinnamic acid	+	Non -IBD
	Linoleoyl ethanolamide	-	IBD:CD
	C14 carnitine	-	IBD:CD+UC
Faecalibacterium prausnitzii	Urobilin	+	Non -IBD
	2-Hydroxyphenethylamine	-	IBD:CD
Eubacterium aligens	Linoleoyl ethanolamide, Palmitoylethanolamide, Docosapentaenoic acid, Eicosatrienoic acid	-	IBD:CD
Bacteroidales bacterium ph8	Urobilin	+	Non -IBD
	C3-DC-CH3 carnitine	-	IBD:CD+UC
Alistipes indistinctus	Dodecanedioic acid	+	Non -IBD
Alistipes senegalensis	Linoleoyl ethanolamide, C18:0 CE	-	IBD:CD
Ruminococcus callidus	Caproic acid	+	Non -IBD
Holdemania filiformis	Urobilin, Cholestenone	+	Non -IBD
	C14 carnitine	-	IBD:CD+UC
Gordonibacter pamelaeae	Pipecolic acid	+	Non -IBD
	Cholate, Chenodeoxycholate	-	IBD:CD
Lachnospiraceae bacterium	Urobilin, Pipecolic acid, 2-Hydroxyhexadecanoate, Cholestenone	+	Non -IBD

		ADMA, Cholate	-	IBD:CD
	Adlercreutzia equolifaciens	Urobilin, Cholestenone, 5α-Cholestanol, 3-Methyladipate-pimelate	+	Non -IBD
		2-Hydroxyphenethylamine, N-Acetylputrescine, ADMA, Cholate, Chenodeoxycholate	-	IBD:CD
		C14 carnitine	-	IBD:CD+UC
	Alistipes onderdonkii	Urobilin, 2-Hydroxyhexadecanoate, Cholestenone, 5α-Cholestanol, Dodecanedioic acid, 3-Methyladipate-pimelate, Undecanedionate, Azelaic acid	+	Non -IBD
		Taurine	-	IBD:CD
		C14 carnitine	-	IBD:CD+UC
	Blautia producta	Docosapentaenoic acid, Eicosatrienoic acid	+	IBD:CD
	Lactobacillus gasseri	Taurine	+	IBD:CD
		Carnosol	-	Non -IBD
	Enterococcus faecium	2-Hydroxyphenethylamine	+	IBD:CD
	Lachnospiraceae bacterium	Docosapentaenoic acid	+	IBD:CD
	Clostridium clostridioforme	Docosapentaenoic acid, Eicosatrienoic acid	+	IBD:CD
		Carnosol, Urobilin	-	Non -IBD
	Roseburia gnavus	Docosapentaenoic acid, Eicosatrienoic acid	+	IBD:CD
		Caprylic acid, 5α-Cholestanol, Caproic acid	-	Non -IBD
[113]	*Clostridium*	Glicyne, Homocysteine	+	IBS
n = 30; HC *n* = 15;	*Lachnospira*	Homocysteine	+	
IBS *n* = 15	*Haemophilus*	Homocysteine	+	
Faeces	*Corynebacterium*	Homocysteine	-	
	Lachnospiraceae	Homocysteine	-	
[114]	*Ruminococcus gnavus* group	Glycoprotein acetyls	+	Steatosis
n = 1355; No-Steatosis *n* = 883; Steatosis *n* = 472 Serum				
[115]	*Gemmiger formicilis*	Lithocholic acid	+	NAFLD

n = 68; HC *n* = 36; NAFLD *n* = 32		7-Dehydrocholic acid, 3-Dehydrocholic acid, Cholic acid, Chenodeoxycholic acid, 12-Dehydrocholic acid, 3β-cholic acid, βMuricholic acid, Allocholic acid, 6-Keto-Lithocholic acid, Ursodeoxycholic acid, Taurohyocholic acid, Tauroursodeoxycholic acid, Chenodeoxycholic acid-3Gln, Taurocholic acid, Glycocholic acid, Glycochenodeoxycholic acid, 6,7-Diketolithocholic acid	-
	Ruminococcus bicirculans	7,12-Diketolithocholic acid, 7-Ketolithocholic acid, Lithocholic acid, Hyodeoxycholic acid, Nordeoxycholic acid	+
	Neglecta timonensis	7,12-Diketolithocholic acid, 7-Ketolithocholic acid, Lithocholic acid, Glycolithocholic acid, βDeoxycholic acid, Nordeoxycholic acid	+
		7-Dehydrocholic acid, 3-Dehydrocholic acid, Cholic acid, Chenodeoxycholic acid, 12-Dehydrocholic acid, Taurohyocholic acid, Chenodeoxycholic acid-3Gln, Taurocholic acid	-
	Oscillibacter sp.	7,12-Diketolithocholic acid, 7-Ketolithocholic acid, Lithocholic acid, Glycolithocholic acid, Dehydrocholic acid, βDeoxycholic acid, Hyodeoxycholic acid, Nordeoxycholic acid	+
		3-Dehydrocholic acid, Cholic acid, Chenodeoxycholic acid, Murocholic acid, Taurocholic acid	-
	Enterobacter cloacae	Chenodeoxycholic acid-3Gln	+
		7,12-Diketolithocholic acid, Hyodeoxycholic acid	-
	Escherichia coli	Glycocholic acid	+
		7,12-Diketolithocholic acid, 7-Ketolithocholic acid, Lithocholic acid, βDeoxycholic acid, Hyodeoxycholic acid, Nordeoxycholic acid	-
	Eubacterium sp.	7,12-Diketolithocholic acid	+
		Glycolithocholic acid-3S, Chenodeoxycholic acid-3Gln, Taurodeoxycholic acid	-
	Bacteroides intestinalis	Taurolithocholic acid, 6,7-Diketolithocholic acid	+

	Akkermansia muciniphila	7,12-Diketolithocholic acid, 7-Ketolithocholic acid, Lithocholic acid, Glycolithocholic acid, Deoxycholic acid, Dehydrocholic acid, βDeoxycholic acid, Glycodeoxycholic acid, αMuricholic acid	-	
	Eubacterium sp.	7,12-Diketolithocholic acid, 7-Ketolithocholic acid, Lithocholic acid	+	
[116]<br>*n* = 115; HC n=54; OB *n* = 8; NAFLD *n* = 27; NASH *n* = 26<br>Faeces	*Ruminococcus*	2-Butanone	-	NAFLD
	Coprococcus	4-Methyl-2-pentanone	-	HC
		2-Butanone	-	NAFLD
	Streptococcus	4-Methyl-2-pentanone	-	HC
	Blautia	4-Methyl-2-pentanone	+	NAFLD
[117]<br>*n* = 201; HC *n* = 40; CAD *n* = 161<br>Serum	*Faecalibacterium*<br>*Roseburia*<br>*Oscillibacter*<br>Lachnospiraceae (CAG4)	Steroids, Phosphatidylethanolamine, Phosphatidylcholine, Ceramides, Glycerophospholipid, Potassium chloride	+	CAD -
		Fatty acyls, Carboxylic acids, Benzene/derivatives, Prenol lipids, Glycerolipids, Amino acids (L-Leucine)	-	CAD +
	Clostridium IV<br>*Alistipes*<br>*Butyricimonas*<br>Clostridiales (CAG5)	Potassium chloride, Addictives/ingredients	-	CAD -
	Escherichia/Shigella<br>*Bacteroides*<br>*Dialister*<br>Lactobacillaceae<br>*Eggerthella*<br>*Clostridium IV, XIVa, XIVb, XVIII*<br>*Flavonifractor*<br>*Coprobacillus*<br>Erysipelotrichaceae incertae sedis	Phosphatidylethanolamine, Phosphatidylcholine	-	CAD -

(CAG9)			
Butyricimonas	Fatty acyls, Carboxylic acids, Glycerolipids	+	CAD +
Bacteroides			
Barnesiella			
Coprobacer	Addictives/ingredients	-	CAD -
Alistipes			
(CAG13)			
Ruminococcus 2	Prenol lipids	-	CAD +
Dorea			
Blautia			
Clostridium XVIII			
Lachnospiracea incertae sedis			
(CAG14)			
Anaerostipes	Sphingolipids, Phosphatidylethanolamine, Addictives/ingredients	+	CAD -
Blautia			
Lactobacillaceae			
Fusicatenibacter	Benzene/derivatives, Glycerolipids	-	CAD +
Clostridium XIVa			
Gemella			
Bifidobacterium	Taurine	-	CAD -
Saccharibacteria genera incertae sedis			
(CAG15)			
Roseburia	Addictives/ingredients	+	CAD -
Clostridium XIVb			
Parasutterella	Fatty acyls, Carboxylic acids, Benzene/derivatives, Prenol lipids, Glycerolipids	-	CAD +
Butyricicoccus			
Lachnospiracea incertae sedis	Taurine	-	CAD -
(CAG16)			
Oscillibacter	Fatty acyl carnitines, Addictives/ingredients	-	CAD -
Clostridium IV			

	(CAG19)			
	Oscillibacter	Benzene/derivatives	+	CAD +
	Clostridium IV, XIVa (CAG23)	Fatty acyl carnitines, Potassium chloride, Addictives/ingredients	-	CAD -
[118] n = 196; HC n = 41; pHT n = 56; HT n = 99 n = 124; HC n = 30; pHT n = 31; HT n = 63 subset Serum	*Butyricicoccus*	PA (12:0/0:0), Hippurin-1	+	HT/HC
		LysoPC (18:2)	-	HC
	Intestinimonas	Nα-Acetyl-L-arginine, S-Carboxymethyl-L-cysteine, Hippurin-1	+	HT/HC/HC
		LysoPC (18:2)	-	HC
	Ruminococcus	Trichloroethanol glucuronide	+	HC
		PA (12:0/0:0), 3-Keto stearic acid, LysoPC (18:2), PS(O-18:0/0:0)	-	HT/HT/HC/HC
	Paenibacillus	9,10-Dichloro-octadecanoic acid, Petunidin 3-rhamnoside 5-glucoside, Hippurin-1	+	HT/HT/HC
		Trichloroethanol glucuronide	-	HC
	Lachnoclostridium	PA (12:0/0:0), 9,10-Dichloro-octadecanoic acid, Petunidin 3-rhamnoside 5-glucoside, 3-Keto stearic acid, Pyridine	+	HT/HT/HT/HT/HC
		Trichloroethanol glucuronide	-	HC
	Butyrivibrio	Petunidin 3-rhamnoside 5-glucoside	+	HT
	Faecalibacterium	Petunidin 3-rhamnoside 5-glucoside, 3-Keto stearic acid	+	HT/HT
	Enterococcus	9,10-Dichloro-octadecanoic acid	+	HT
	Coprococcus	Nα-Acetyl-L-arginine, Petunidin 3-rhamnoside 5-glucoside	+	HT/HT
	Flavonifractor	3-Keto stearic acid	-	HT
	Blautia	PS (O-18:0/0:0)	+	HC
		PA (12:0/0:0), 9,10-Dichloro-octadecanoic acid, 3-Keto stearic acid	-	HT/HT/HT
	Bifidobacterium	Trichloroethanol glucuronide	+	HC
		PA (12:0/0:0), 9,10-Dichloro-octadecanoic acid, 3-Keto stearic acid	-	HT/HT/HT
	Subdoligranulum	LysoPC(18:2), Trichloroethanol glucuronide	+	HC/HC
		PA (12:0/0:0), 9,10-Dichloro-octadecanoic acid, Petunidin 3-rhamnoside 5-glucoside	-	HT/HT/HT
	Marvinbryantia	S-Carboxymethyl-L-cysteine, Trichloroethanol glucuronide	+	HC/HC

	PA (12:0/0:0), 9,10-Dichloro-octadecanoic acid, Petunidin 3-rhamnoside 5-glucoside	-	HT/HT/HT
Holdemania	Nα-Acetyl-L-arginine, Pyridine, S-Carboxymethyl-L-cysteine, PS (O-18:0/0:0)	+	HT/HC/HC/HC
	PA (12:0/0:0)	-	HT
Robinsoniella	Nα-Acetyl-L-arginine, Pyridine, PS(O-18:0/0:0), Trichloroethanol glucuronide	+	HT/HC/HC/HC
Veillonella	3-Keto stearic acid, LysoPC(22:5), Pyridine	+	HT/HC/HC
	PA (12:0/0:0)	-	HT
Clostridium	9,10-Dichloro-octadecanoic acid	-	HT
Oribacterium	Pyridine	+	HC
	PA (12:0/0:0), Nα-Acetyl-L-arginine, 3-Keto stearic acid, PS (O-18:0/0:0)	-	HT/HT/HT/HC
Bilophila	Pyridine	+	HC
	Nα-Acetyl-L-arginine, 3-Keto stearic acid	-	HT/HT
Akkermansia	PA (12:0/0:0), Pyridine, Trichloroethanol glucuronide	+	HT/HC/HC
	Nα-Acetyl-L-arginine	-	HT
Pyramidobacter	LysoPC (22:5), Hippurin-1	+	HC/HC
	PA (12:0/0:0), Pyridine	-	HT/HC
Tyzzerella	LysoPC (18:2)	+	HC
	Petunidin 3-rhamnoside 5-glucoside, 3-Keto stearic acid, Pyridine	-	HT/HT/HC
Roseburia	LysoPC (22:5), S-Carboxymethyl-L-cysteine, Hippurin-1	+	HC/HC/HC
	PA (12:0/0:0), 9,10-Dichloro-octadecanoic acid, Nα-Acetyl-L-arginine	-	HT/HT/HT
Ruminiclostridium	LysoPC (22:5), LysoPC (18:2), Hippurin-1	+	HC/HC/HC
	Nα-Acetyl-L-arginine, Trichloroethanol glucuronide	-	HT/HC
Oscillibacter	PA (12:0/0:0)	+	HT
	Nα-Acetyl-L-arginine, LysoPC (22:5), S-Carboxymethyl-L-cysteine	-	HT/HC/HC
Treponema	PA (12:0/0:0)	+	HT
	LysoPC (22:5), Pyridine, LysoPC (18:2)	-	HC/HC/HC
Peptoclostridium	PA (12:0/0:0), 9,10-Dichloro-octadecanoic acid	+	HT/HT

	S-Carboxymethyl-L-cysteine, Trichloroethanol glucuronide, Hippurin-1	-	HC/HC/HC
Anaerotruncus	PA (12:0/0:0), 9,10-Dichloro-octadecanoic acid, PS (O-18:0/0:0), Hippurin-1	-	HT/HT/HC/HC
Eggerthella	3-Keto stearic acid, LysoPC (22:5)	+	HT/HC
	PA (12:0/0:0), 9,10-Dichloro-octadecanoic acid, Hippurin-1	-	HT/HT/HC
Acidiphilium	PA (12:0/0:0), 9,10-Dichloro-octadecanoic acid, 3-Keto stearic acid, LysoPC(18:2), PS(O-18:0/0:0), Trichloroethanol glucuronide	+	HT/HT/HT/HC/HC/HC
	Nα-Acetyl-L-arginine, Petunidin 3-rhamnoside 5-glucoside	-	HT/HT
Klebsiella	9,10-Dichloro-octadecanoic acid, S-Carboxymethyl-L-cysteine	+	HT/HC
	Hippurin-1	-	HC
Azospirillum	Nα-Acetyl-L-arginine	+	HT
	Hippurin-1	-	HC
Prevotella	PA (12:0/0:0), 9,10-Dichloro-octadecanoic acid	+	HT/HT
	3-Keto stearic acid, LysoPC (22:5), Trichloroethanol glucuronide	-	HT/HC/HC
Dorea	PA (12:0/0:0), 3-Keto stearic acid	+	HT/HT
	9,10-Dichloro-octadecanoic acid, Trichloroethanol glucuronide	-	HT/HC
Acidaminococcus	PA (12:0/0:0)	+	HT
	Trichloroethanol glucuronide	-	HC
Enterobacter	PA (12:0/0:0), 9,10-Dichloro-octadecanoic acid, Petunidin 3-rhamnoside 5-glucoside	+	HT/HT/HT
	3-Keto stearic acid	-	HT
Streptococcus	Hippurin-1	+	HC
	Nα-Acetyl-L-arginine, LysoPC (18:2)	-	HT/HC
Porphyromonas	9,10-Dichloro-octadecanoic acid, LysoPC (22:5), Pyridine, Hippurin-1	+	HT/HC/HC/HC
	PS (O-18:0/0:0)	-	HC
Coprobacillus	Petunidin 3-rhamnoside 5-glucoside	+	HT
	9,10-Dichloro-octadecanoic acid, LysoPC (22:5), S-Carboxymethyl-L-cysteine	-	HT/HC/HC
Actinomyces	3-Keto stearic acid	+	HT

	PA (12:0/0:0), 9,10-Dichloro-octadecanoic acid, LysoPC (22:5), Pyridine	-	HT/HT/HC/HC
Desulfovibrio	3-Keto stearic acid	+	HT
	Pyridine, Trichloroethanol glucuronide, Hippurin-1	-	HC/HC/HC

CAD: coronary artery disease; CAG: co-abundance group; CD: Crohn´s disease; HC: healthy control; HCHS/SOL: Hispanic Community Health Study/Study of Latinos; HT: hypertension; IBD: irritable bowel disease; IBS: irritable bowel syndrome; IGR: impaired glucose regulation; LC: lean control; LH: lean healthy; MLGs: metagenomic linkage groups; MM: microbial modules; NAFLD: non-alcoholic fatty liver disease; NASH: non-alcoholic steatohepatitis; ND: not determined; NGT: normal glucose tolerance; Non-PN SBS: parenteral nutrition-independent short bowel syndrome; NW: normal weight; OB: obese; ObH: obese healthy; OW: overweight; PCOS: polycystic ovary syndrome; p-HT: pre-hypertension; SBS I: parenteral nutrition-dependent short bowel syndrome I; SBS II: parenteral nutrition-dependent short bowel syndrome II; T2D: type 2 diabetes; T2D+: diabetes with chronic complications; T2D-: diabetes without chronic complications; UC: ulcerative colitis.
